# Therapeutic approaches targeting CD95L/CD95 signaling in cancer and autoimmune diseases

**DOI:** 10.1038/s41419-022-04688-x

**Published:** 2022-03-17

**Authors:** Vesna Risso, Elodie Lafont, Matthieu Le Gallo

**Affiliations:** 1 INSERM U1242, Oncogenesis Stress Signaling, University of Rennes, Rennes, France; 2 Centre de lutte contre le cancer Eugène Marquis, Rennes, France

**Keywords:** Drug development, Apoptosis

## Abstract

Cell death plays a pivotal role in the maintenance of tissue homeostasis. Key players in the controlled induction of cell death are the Death Receptors (DR). CD95 is a prototypic DR activated by its cognate ligand CD95L triggering programmed cell death. As a consequence, alterations in the CD95/CD95L pathway have been involved in several disease conditions ranging from autoimmune diseases to inflammation and cancer. CD95L-induced cell death has multiple roles in the immune response since it constitutes one of the mechanisms by which cytotoxic lymphocytes kill their targets, but it is also involved in the process of turning off the immune response. Furthermore, beyond the canonical pro-death signals, CD95L, which can be membrane-bound or soluble, also induces non-apoptotic signaling that contributes to its tumor-promoting and pro-inflammatory roles. The intent of this review is to describe the role of CD95/CD95L in the pathophysiology of cancers, autoimmune diseases and chronic inflammation and to discuss recently patented and emerging therapeutic strategies that exploit/block the CD95/CD95L system in these diseases.

## Introduction

The division, differentiation, and death of a cell are highly regulated events in every developing organism and, in the adult individual, the loss of single cells plays a primary role in the maintenance of tissue homeostasis. Cell death, considered as a physiological event, can be defined as a highly evolved and conserved cell elimination mechanism, which responds to homeostatic and morphogenetic stimuli. The cells have a genetically-encoded death program that is finely controlled at the transcriptional and post-transcriptional levels. The definition of “programmed or regulated cell death” (RCD) is appropriate for the description of this phenomenon. Amongst the different types of RCD [1], apoptosis remains the most studied. Two major apoptotic pathways have been described: the extrinsic pathway or Death Receptor (DR) pathway and the intrinsic or mitochondrial pathway, which are linked [2]. In both pathways, specific aspartyl cysteine proteases (caspases) are activated and cleave cellular substrates, ultimately leading to the disruption of multiple cellular processes and morphological changes, such as cell shrinkage or the formation of apoptotic bodies, typical of apoptosis. The crosstalk between the two apoptotic pathways is carried out by the fact that caspase-8, involved in the extrinsic pathway, is able to cleave BID, a Bcl-2 family protein involved in the intrinsic pathway, thus activating the latter after apoptotic stimulus via DR and eventually strengthening the apoptotic signal [3–5].

### Molecular bases of apoptotic signaling

#### The intrinsic mitochondrial-mediated apoptotic pathway

The intrinsic or mitochondrial pathway can be triggered by a variety of cellular stressors (e.g DNA-damaging agents, nutrient deprivation, hypoxia) and is tightly controlled by pro- and anti-apoptotic members of the Bcl-2 family of proteins. These cellular stress primarily lead to the increased transcription and/or post-translational activation of pro-apoptotic members of the Bcl-2 family of proteins [6, 7]. The key event of this intrinsic RCD is the mitochondrial outer membrane permeabilization (MOMP) induced by the oligomerization of the pro-apoptotic effector members of this family (BAX, BAK, and in some cases BOK) at the MOM [8]. MOMP allows the release of several caspase activators, such as the cytochrome c, from the mitochondrial intermembrane space to the cytosol. Hence, understanding the molecular bases of the pore-forming capacity of the effectors and of the regulation of their activation is crucial [8–11]. In the cytosol, cytochrome c promotes the assembly of a caspase activation platform called the apoptosome that also includes caspase-9, the activation factor of apoptotic proteases-1 (Apaf-1) and dATP [12]. Indeed, in the absence of apoptotic stimuli, Apaf-1 exists in an inactive monomeric conformation while it undergoes heptameric oligomerisation upon binding to cytochrome c and dATP in apoptotic conditions [13]. The formation of the apoptosome triggers the activation of caspase-9 which in turn activates the effector caspases-3, -7 that drive cell demise [14, 15]. MOMP also promotes the release of anti-apoptotic factors, such as the second mitochondrial activators of caspase (Smac/Diablo) and Omi/HtrA2 (high temperature requirement A2) and endonuclease G (EndoG) [16]. The protein Smac [17, 18] interacts with the BIR2 and BIR3 domains of the X-linked inhibitor of apoptosis protein (XIAP), neutralizing the inhibitory effect of XIAP on caspases-3, 7, and 9 [19]. Omi/HtrA2 [20–23] is a serine protease which, once released into the cytosol, is also able to significantly increase the activity of caspases by inhibiting XIAP. Noteworthy, MOMP can also induce non-apoptotic cell death such as ferroptosis, necroptosis and pyroptosis as recently reviewed elsewhere [7, 24].

The extent of MOMP largely defines the propensity of a cell to die or survive upon cell stress. The availability and activity of the different Bcl-2 family members influences the cellular readiness or “priming status” for MOMP. This priming status can be determined through BH3-profiling [25, 26] that evaluates MOMP upon incubation of permeabilized cells with BH3 peptides mimicking the action of some pro-apoptotic members of the Bcl-2 family. This assay has mainly been used to predict the sensitivity of cancer cells to various chemotherapeutic agents (resistant cells usually display lower priming) and to interrogate the sensitivity of cancer cells to the increasing arsenal of BH3-mimetics (molecules mimicking the activity of some pro-apoptotic Bcl-2 family members). MOMP is not necessarily a complete process. Indeed, partial MOMP has been observed when apoptotic induction is weak (minority MOMP) or accompanied by caspase inhibition (incomplete MOMP). The ability of cells to retain some non-permeabilized mitochondria, ATP synthesis and to eliminate damaged mitochondria influences their propensity to survive upon incomplete MOMP. Indeed, the remaining intact mitochondria can repopulate the whole mitochondrial pool [27, 28]. In the case of minority MOMP, caspase activation is insufficient to drive death but can promote DNA damage and genomic instability [29]. In addition, several reports indicate that MOMP can initiate multiple inflammatory signaling, for example the cGAS/STING [30, 31] or the NF-κB pathways [32]. Thereby MOMP can impact on the cell and its microenvironment beyond its ability to promote cell death.

Taken together, it appears that further understanding the mechanisms dictating the extent of MOMP, its ability to induce various types of cell death as well as non-death pathways in different pathophysiological contexts (e.g., upon pathogen infection, during tumor progression, etc.) and in different cell types will be required to fully expand the therapeutic targeting of the mitochondrial pathway. For further considerations on this topic, we advise readers to explore the many recent reviews available [7, 24, 33].

#### CD95 and CD95L: main structural features

**Fig. 1:**
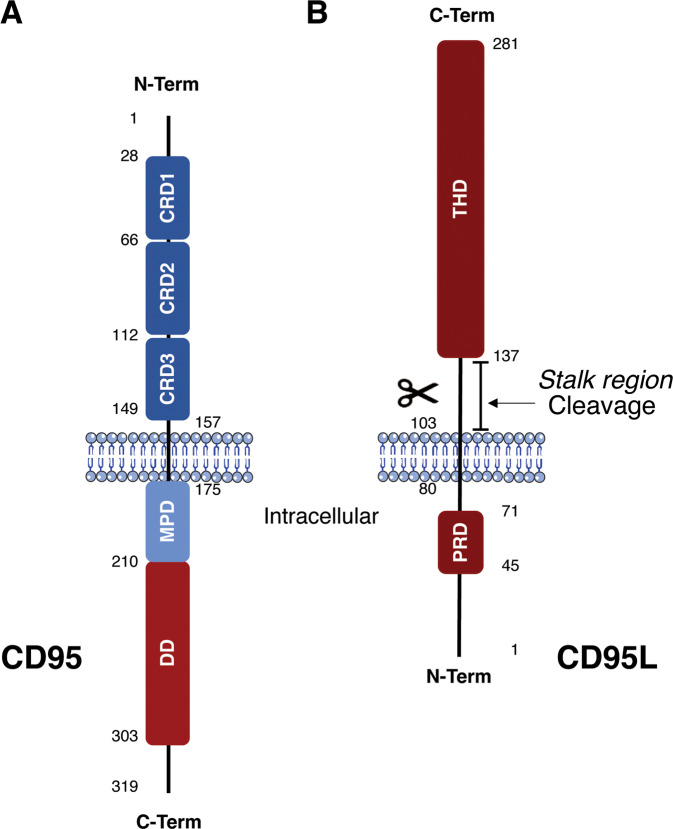
The CD95 receptor and its cognate ligand CD95L. Schematic representation of the functional domains of the CD95 Death Receptor (**A**) and its ligand CD95L in its membrane-bound form (**B**). (DD Death Domain, MPD Membrane Proximal Domain, CRD Cysteine-Rich Domain, PRD Proline-Rich Domain, THD TNF Homology Domain).

The extrinsic apoptosis pathway takes its name from the extracellular signal molecules that bind to receptors exposed on the surface of target cells, leading to a different way of activating the apoptotic signal compared to the mitochondrial-mediated one. There is a family of receptors specialized in the transmission of the signal upon binding by their cognate ligand that leads to the extrinsic programmed cell death: the DR. The DR belong to the Tumor Necrosis Factor Receptor (TNFR) superfamily, which counts a total of 29 receptors associated with a smaller selection of 19 ligands of the corresponding TNF ligands superfamily. CD95, TNFR1, DR3, DR4, DR5, and DR6 are the most studied DR that, upon ligand binding, convey death signal by using a conserved intracellular region of ~80 amino acids called the “Death Domain” (DD) [34]. This review particularly focuses on the DR CD95, its physiological ligand CD95L and the current approaches developed to therapeutically target this pair. CD95, encoded by the *FAS* gene, is a 319aa type I glycoprotein devoid of enzymatic activity that signals through protein-protein interaction. Mature CD95 is composed of three cysteine-rich extracellular domains, CRD3, CRD2, and CRD1 starting from the transmembrane domain and moving towards the N-Terminal. CRD2 and partly CRD3 are used for the recognition and binding of the ligand, while CRD1, comprising a subdomain called PLAD (Pre-Ligand Assembly Domain) [35, 36], is needed for the preassembly of CD95 in homodimeric or homotrimeric forms at the plasma membrane. The cytosolic region is composed of the previously mentioned Death Domain (DD) [34], which is essential for the transduction of the apoptotic signal, and a Membrane Proximal Domain (MPD) which conveys non-apoptotic signaling (Fig. 1) [37]. CD95L, encoded by the *FASLG* gene, consists of a total of 281aa, an extracellular region with a C-terminus and an intracellular region with an N-terminus. This protein is expressed at the plasma membrane in the form of a homotrimer thanks to the preassembly between monomers that takes place through an extracellular domain called TNF Homology Domain (THD) [38]. The THD also mediates receptor binding. The membrane-proximal extracellular stalk region is proteolytically processed by several metalloproteases to release soluble forms of CD95L (sCD95L), which generally display non-apoptotic activities (see part 2). The cytosolic region is then composed of an 80 amino acid tail containing a domain rich in proline, which is involved in the reverse signaling induced by CD95L–CD95 interaction in CD4 and CD8 T cells (Fig. 1). This reverse signaling involves the co-engagement of the TCR and co-stimulatory receptors along that of CD95/CD95L [39–42]. The reported outcomes of this reverse signaling depends on the cell type, with both proliferation and cell cycle arrest being reported, but the knowledge on this subject is still very partial.

#### Molecular bases of CD95-induced apoptotic signaling

**Fig. 2:**
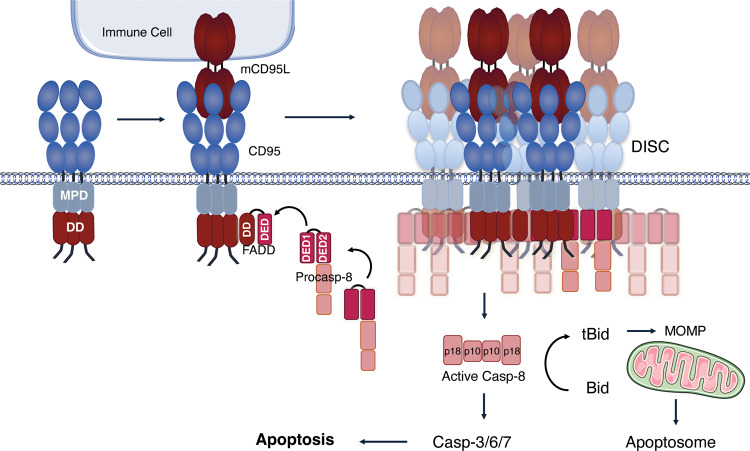
CD95-dependent apoptotic signaling. Representation of the CD95-mediated conventional or apoptotic pathway. The interaction between CD95 and its membrane-bound ligand mCD95L, triggers the recruitment of the adaptor protein FADD, which then recruits procaspase-8 generating the oligomerized DISC. The oligomerisation and auto-cleavage of procaspase-8 into its active form induces then the activation of the effector caspases-3, -6, -7 leading to apoptosis. Active caspase-8 is also able to cleave Bid, generating t-Bid that promotes Mitochondrial outer membrane permeabilization (MOMP) and thus the apoptosome-mediated effector caspase activation.

CD95-mediated extrinsic apoptotic signaling begins with the binding of CD95L, via its THD on CRD2 and part of the CRD3 of CD95. In addition to the pre-association of CD95 mediated by the PLAD [35, 36], Fu et al. recently showed that proline motifs in the transmembrane (TM) domain also contribute to the trimerization of the receptor. Mutations of these motifs did not abrogate PLAD-mediated preassembly of unliganded CD95 but reduced CD95L-induced apoptosis, implying that these residues are important for stabilizing signaling-active CD95 oligomers [43]. Binding of CD95L has been proposed to trigger a reorganization of CD95 multimers and a conformational change in CD95 intracellular domain, allowing for the recruitment of the adaptor FADD (Fas-associated protein with Death Domain) to CD95 via DD-mediated homotypic interactions [44–47]. FADD is necessary for CD95L-induced apoptosis [48, 49]. In addition to its DD, FADD contains a Death Effector Domain and acts as a pivot for the assembly of DED filaments which are chains of proteins formed through DED-mediated interactions [50–52]. The DED chains nucleate from FADD [51–54] and also comprise procaspase-8 and cellular FLICE-like inhibitory proteins (c-FLIP) which are both key players in the cell death network [51, 52]. Extensive work has been undertaken, mainly in the past 15 years, to understand the mode of assembly of these structures. Beyond CD95- and TRAIL-R1/2-associated complexes, similar structures likely also nucleate from other death-inducing complexes such as the ripoptosome, inflammasomes, TNF-induced complex II, as well as the panoptosome [53, 55–57] and could thus influence cell fate upon a plethora of signals. In the case of CD95 signaling, the complex formed by CD95, FADD, caspase-8 and cFLIP constitutes a platform for caspase-8 activation which was first called the DISC (for Death-Inducing Signaling Complex) [44]. Procaspase-8 contains two DEDs, DED1, and DED2, located at its N-terminus and C-terminal large (p18) and small (p10) catalytic subunits. As described below, the formation of the DED filaments allows for the activation of caspase-8 which occurs via dimerization and a serie of internal cleavages, leading to the separation of the tandem DED from the catalytic subunits p18 and p10 [53, 54, 58–60]. The active p10 and p18 subunits are released into the cytoplasm to form mature active caspase-8 (Fig. 2). Fully matured caspase-8, an heterotetramer of two p18 and two p10, cleaves effector caspases-3, 6 and 7, which then cleave sub-cellular substrates, ultimately inducing cell death [61]. Three isoforms of cFLIP have been described: cFLIP long, short and related (cFLIP_L,_ cFLIP_S_ and cFLIP_R_). cFLIP_S_ and cFLIP_R_ comprise solely two tandem DED. In addition to the tandem DEDs, cFLIP_L_ comprises a small and a large caspase-like catalytically inactive subunit. The initial DED-chain model, described by Inna Lavrik and Marion MacFarlane’s laboratories, proposed a nucleation of the chain from FADD involving an interaction between the DED of FADD with the DED1 of caspase-8, whilst further chain elongation implicated an interaction between the DED2 of FADD-associated caspase-8 with the DED1 of the incoming caspase-8, ultimately bringing the two catalytic domains of caspase-8 in close proximity [51, 52, 62, 63]. The molecular configuration of the DED filaments was further unveiled in 2016, by cryogenic electron microscopy (cryo-EM) analysis [53]. This study established that the orientation of the DED filaments actually relies on three different types of interactions (type I, II and III) between DEDs. Rather than a single linear chain nucleating from FADD through type I interactions, three strands of DED chains assemble via type II and III interactions to ultimately form a triple-helical structure [53, 54]. These different types of interactions define a hierarchy in the formation of the DED filaments, with FADD being rather poorly able to nucleate the DED of cFLIP, arguing against the theory of competition between procaspase-8 and c-FLIP for FADD. Thus, by affecting the conformation of caspase-8 and bringing in proximity the catalytic subunits of two procaspase-8, the DED-chain architecture works as a platform for the activation of this initiator caspase [64, 65].

With regard to cFLIP proteins, it was first thought that these act by competing with caspase-8 for FADD binding or by preventing FADD self-association, akin to the viral FLIP MC159 [65], but this view has been challenged. Multiple evidence now demonstrate that cFLIP_S/R_ actually precludes caspase-8 activation within the DISC. Indeed, reports highlighted that cFLIP_S/R_ could limit DED-chain elongation and that cFLIP_S/R_ incorporation into DED filaments actively prevented the formation of inter-strand assembly of caspase-8 catalytic domains [54, 63, 65]. Contrary to the small cFLIP isoforms, cFLIP_L_ has been reported to possess a dual function, promoting or limiting caspase-8 activation and apoptosis. This is likely due to the fact that the cFLIP_L_/caspase-8 heterodimer does possess a catalytic activity, albeit DISC restricted, and that cFLIP_L_ does not limit but promotes DED elongation. Hence, depending on the relative cellular amount of cFLIP_L_ to caspase-8, cFLIP_L_ might either facilitate the formation of filaments, and thereby of apoptosis-inducing caspase-8 homodimers (low cFLIPL to caspase-8 ratio) or, on the contrary (high cFLIP_L_ to caspase-8 ratio), mainly result in formation of cFLIP_L_/caspase-8 heterodimers which, whilst able to cleave local substrates (e.g RIPK1), do not mediate apoptosis [63, 66–70].

Another initiator caspase, caspase-10, can be recruited to the TRAIL-R1/2 and CD95 DISC [66, 71, 72]. The role of this caspase in apoptosis induction by CD95L and TRAIL, and in particular its ability to substitute to caspase-8 loss, has been controversed. Caspase-10 is conserved in multiple other vertebrates [73] but lost in certain rodents (mice and rats) which has limited the study of its in vivo function. Some studies, mainly but not exclusively using Jurkat cells or primary T cells, reported that caspase-10 can contribute to DR-induced apoptosis, sometimes independently of caspase-8 [71, 74–79]. Interestingly, a recent study argued that this protease displays anti-apoptotic properties in certain cell lines [80]. Of note, this initiator caspase has been found as different splice variants in human cells, which have also been suggested to display opposing functions towards DR-mediated apoptosis [81]. How each of these isoforms and potentially their post-translational modifications (PTMs) impact on the DED-triple helix formation remains to be deciphered. Indeed, PTMs, most prominently glycosylation, phosphorylation and ubiquitination, of core components of the DISC proteins represent additional crucial checkpoints of DR signaling [82–84].

As mentioned above, caspase-8 also cleaves Bid, generating t-Bid that promotes MOMP and thus apoptosome-mediated effector caspase activation. Whether the engagement of the mitochondrial pathway downstream of CD95 is required for completion of apoptosis depends on the multiple variables described to influence DISC formation (e.g expression level of the DISC components, local lipid composition of the plasma membrane, etc.) as well as downstream regulators of the apoptosis pathway such as XIAP [85–87]. The discovery that caspase-8 is essential during embryonic development lead to the identification of its role as a regulator of necroptosis. Indeed, caspase-8, in concert with cFLIP_L_, is able to cleave RIPK1, along other key components of the necroptotic cascade, which limits necroptosis induction, as reviewed in [61]. In addition, as further developed later, several of the players of the apoptotic pathway, and in particular DISC components, are also involved in non-cytotoxic signaling outputs.

## Involvement of CD95/CD95l in cancer and autoimmune diseases

### Cancer

Multiple defects in the DR-mediated pathway have been observed in human tumors [88–91]. In healthy individuals, extrinsic apoptosis plays a central role in the immune-mediated elimination of infected or transformed cells. Therefore, defects in the extrinsic apoptotic pathway contribute to tumorigenesis primarily by limiting the efficiency of immune surveillance [92]. Cancer cells have different ways of escaping from apoptosis [93]. These include modification of the expression of pro- and anti-apoptotic proteins, such as inhibitors of apoptosis (IAPs) and the anti-apoptotic members of the Bcl-2 family among others, as well as the expression of CD95 itself at the membrane [94, 95]. Mutations in the *FAS* gene have been detected in both hematologic and solid tumor malignancies [96–99]. These mutations are mainly located in exon 8 and 9, which code for the DD, thus leading to resistance to CD95-mediated apoptosis [91, 93]. Accumulating evidence has shown that CD95 signaling cascades are often disrupted in several autoimmune diseases and malignant tumors [100–102], leading to the triggering of pro-tumorigenic cellular outcomes, rather than apoptosis [89, 103]. Considering the potential pro-tumorigenic effect of an incomplete induction of mitochondria-dependent death-signaling mentioned above, one could hypothesize that weak apoptotic signaling downstream of CD95 could also have tumor-promoting effects. Furthermore, the quality of cell death induced downstream of CD95 might also differentially impact on inflammation and tumor progression, even though this remains to be tested. In addition, several non-apoptotic pathways are also induced by CD95L, as detailed below, and contribute to its tumor-promoting and pro-inflammatory roles [88].

### Non-apoptotic CD95-mediated pathways (NF-κB, MAPK, PI3K/Akt)

#### NF-κB pathway

Several studies reported that CD95-mediated stimulation can induce the apoptotic pathway in some cells, while in others, the non-apoptotic NF-κB (nuclear factor kappa B) pathway is favored [104, 105]. NF-κB is a transcription factor playing an important role in the inflammatory responses as well as in the regulation of cell survival, differentiation and proliferation. A non-optimal regulation of this signaling pathway has been associated with a high incidence of pathological conditions, such as cancer and chronic inflammation [106]. At the cell population level, the stimulation of CD95 by CD95L has long been reported to concomitantly induce apoptotic signaling and NF-κB activation [105, 107]. More recently, single cell studies have assessed if the apoptotic and NF-κB pathways were activated in the same cell [107, 108]. NF-κB was found to be activated in dying apoptotic cells, confirming the hypothesis that CD95-mediated NF-κB activation is correlated with the production of the so-called “find and eat me” pro-inflammatory cytokines, including IL-6, IL-8, CXCL1, MCP-1, and GMCSF [104]. Some of these cytokines act as chemokines and are therefore able to affect the tumor immune microenvironment.

**Fig. 3:**
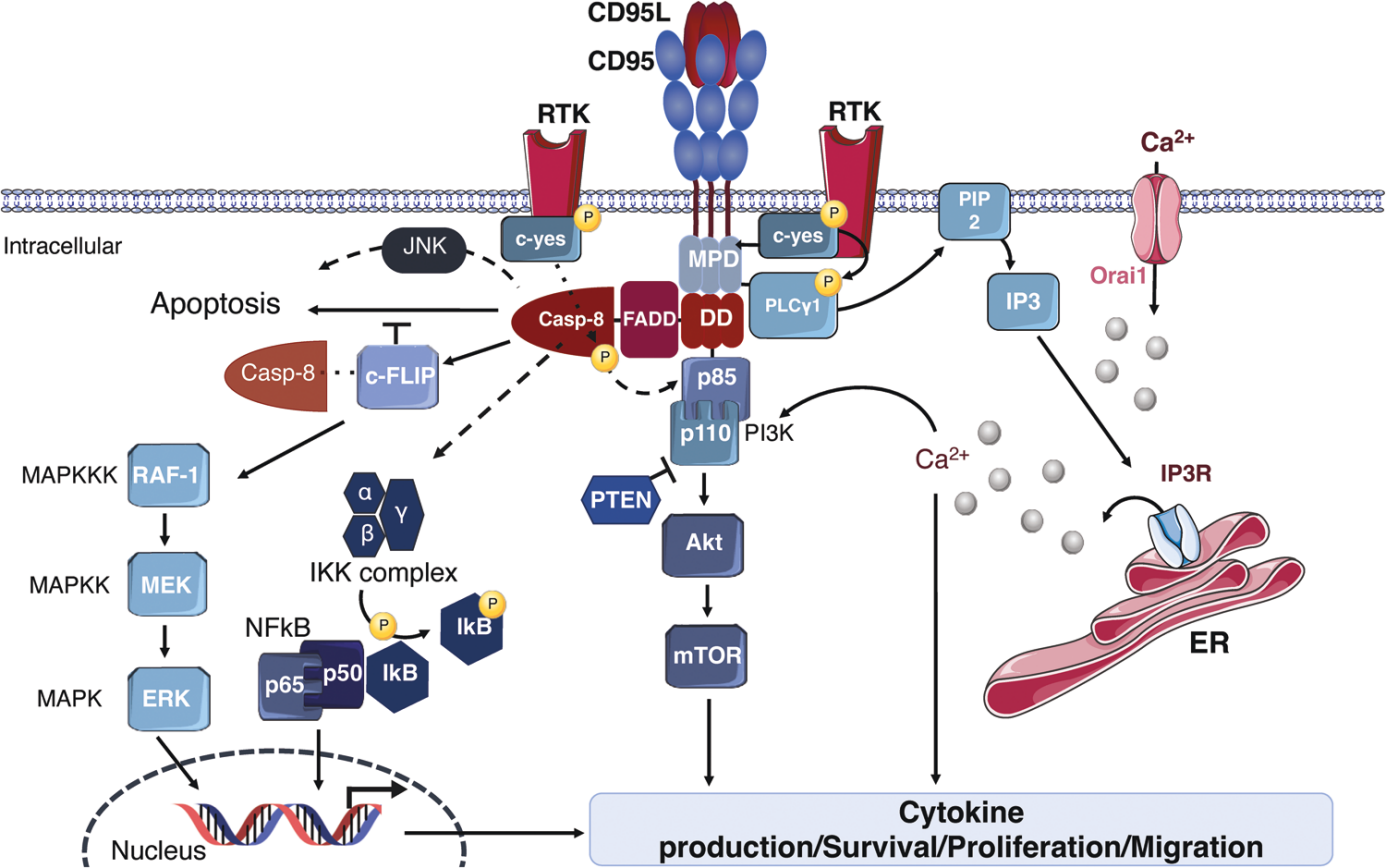
CD95-dependent non-apoptotic signaling. Representation of the CD95-mediated unconventional or non-apoptotic pathways. The interaction between CD95 and its ligand CD95L recruits several adaptor proteins leading to the activation of the MAPK, NF-κB and PI3K pathways. The MAPK pathway requires a cascade of phosphorylations to eventually activate ERK, allowing its translocation to the nucleus where it induces the transcription of pro-survival/proliferation/pro-inflammatory genes. The NF-κB heterodimer is kept inactive by IκB, which after IKK-mediated phosphorylation releases NF-κB allowing its translocation to the nucleus where it promotes the transcription of pro-inflammtory/proliferation/migration genes. The PI3K/Akt and PLCy1 pathways are functionally linked in triggering the cell migration. Active PLCy1 participates in the elevation of cytoplasmic calcium levels, which then leads the activation of biochemical pathways that leads to cell proliferation, survival and migration through the phosphorylation and activation of Akt.

Mechanistically, it appears that CD95 mediates NF-κB activation through a FADD and caspase-8-involving pathway [104, 109–111]. The Death Domain of CD95, FADD, and caspase-8 were in fact reported as required for NF-κB activation by CD95L [110]. Experiments carried out inhibiting caspases prevented TRAIL/anti-APO-1-induced apoptosis, but not NF-κB activation, indicating that both pathways bifurcate upstream of caspase-8 full activation [112]. Furthermore, the ability of DR to induce NF-κB activation was drastically reduced in a FADD-deficient CD95^pos^ cell line (e.g., Jurkat cells) [112]. Caspase-8 participates in CD95L- and TRAIL-induced inflammatory signaling as a scaffold for assembly of a Caspase-8-FADD-RIPK1-containing complex, leading to NF-κB-dependent inflammation [109, 113]. Whilst this has not been studied for CD95 yet, it is tempting to speculate that NF-κB activation could also be ignited from the CD95 DISC, as recently shown for TRAIL [114]. Contrary to FADD and caspase-8 which seem to be essential for NF-KB activation upon CD95L, the role of RIPK1 in this process seems to be less pronounced and depends on the cell type [104, 109]. Recently, Horn et al. described a new role for caspase-10 that would negatively regulate the caspase-8-induced cell death, thus activating the cell survival induced by the NF-κB pathway [80]. TRADD, which is essential for the TNF-alpha-induced NF-κB activation, was not involved in the CD95L-induced NF-κB activation [110]. Experiments performed on cell lines resistant to CD95-mediated apoptosis, reported TRAF2 as a key player in pancreatic cancer pathophysiology [115]. This group also observed that the stimulation of TRAF2-overexpressing cells with CD95L led to induction of NF-κB, enhanced IL-8-secretion, and a further increased invasiveness. In fact, several E3 ligases contribute to NF-κB activation upon CD95 stimulation, namely cIAP1/2 and the Linear UBiquitin chain Assembly Complex (LUBAC), likely in a manner similar to their roles in TNF and TRAIL-induced gene-activation [104, 114, 116]. Downstream of these different actors, the activation of NF-κB relies on IκBα degradation, the protein responsible for constitutively inhibiting NF-κB. In a manner similar to TNF and TRAIL signaling, it is likely that several components of the CD95 DISC and/or secondary complex modified with ubiquitin allow the recruitment and activation of the IKK complex and potentially the TAB/TAK1 complex. The IKK complex is composed of three subunits (i.e., IKKα, IKKβ, IKKγ). The IKKβ subunit can then phosphorylate IκBα, marking it for lysine-48 ubiquitination and degradation by the proteasome. This leads to the translocation of NF-κB into the nucleus which promotes the expression of multiple genes including pro-inflammatory cytokines as well as anti-apoptotic proteins, such as cIAP1, cIAP2, and XIAP (Fig. 3) [117, 118]. Moreover cFLIP can be upregulated in some cell lines under critical involvement of the NF-κB pathway [119, 120] also resulting in increased resistance to CD95L or TNF.

#### MAPK pathway

The MAPK family includes six main groups in humans, among which JNK (Jun N-terminal Kinase), ERK1/2 and the p38 isoform must be mentioned for their involvement in CD95-mediated pro- and anti-apoptotic signaling pathways [121–123]. The induction of the mitogen-activated protein kinase/extracellular signal-regulated kinase (MAPK/ERK) signaling pathway, which regulates growth, proliferation, differentiation, survival, innate immunity and cellular development is involved in tumorigenesis in multiple tumor types [124]. In the latent state, the inactive MAPKs are cytosolic. The activation of the different MAPKs takes place according to a common general scheme, which provides for a series of sequential phosphorylations catalyzed by different kinases activated in succession. MAPK is phosphorylated by a MAPK kinase (MKK), itself phosphorylated by a MAPK kinase kinase (MKKK), in turn activated by an activator protein.

CD95-mediated stimulation has been suggested early on to induce the JNK pathway through the DAXX adapter protein (Death domain associator protein 6), which after fixation with CD95-DD induces the apoptotic pathway [125, 126]. c-FLIP can block this pathway by inhibiting DAXX [127]. CD95-mediated JNK activation also appears to occur rather slowly, compared to other cell stress stimuli, such as inflammatory cytokines and oxidative stress [128]. Indeed, expression of cFLIP variants or use of different caspase inhibitors in primary human keratinocytes, blocked late death ligand-induced JNK or p38-MAPK activation, suggesting that these responses are secondary to caspase activation [129]. This may be due to the fact that caspase-3 can cleave and thereby activate the MAP3K MEKK1 [128]. Of note, the signal induced by soluble CD95L rather results in a rapid and transient phosphorylation of ERK1/2 [130]. This MAPK protein is widely involved in enhancing growth and proliferation upon CD95 activation [121]. Stimulation of CD95 on primary sensory neurons triggers neurite growth through sustained activation of the extracellular signal-regulated kinase (ERK) pathway and subsequent upregulation of p35, a neurite growth mediator [131]. Of note, in pancreatic apoptosis-resistant tumor cells, CD95L- and TRAIL-induced upregulation of pro-inflammatory genes was found to be partially depend on the ERK signaling pathway via caspase-mediated activation [132]. The same group suggested that the stimulation of the ERKs pathway must probably depend on a caspase-dependent factor operating downstream of the DISC complex. According to another group, CD95L can also induce the autocrine production of EGFR (Epidermal Growth Factor Receptor) ligands and the consequent activation of EGFR followed by ERK1 and ERK2 mitogen-activated protein kinases [133]. In primary fetal astrocytes, blocking ERK phosphorylation with specific inhibitors resulted in a significant reduction of CD95-induced proliferation [134]. In this context, ERK phosphorylation is also caspase-dependent. Noteworthy, cFLIP_L_ can also contribute to ERK activation. Indeed, caspase-8 can cleave cFLIP_L_ into different cleavage products. One of these cleavage products is identified with the name of p43-FLIP [135], which associates with Raf-1 activating the phosphorylation cascade, leading to ERK activation and ultimately to ERK translocation into the nucleus, where it exerts proliferative or pro-inflammatory effects through downstream transcription factor targets (Fig. 3).

#### PI3K pathway

As mentioned previously, mCD95L can be cleaved by various metalloproteases to produce several soluble forms of the ligand, together referred here as sCD95L [136–140]. Soluble CD95L has been shown to be accumulated in the serum of patients suffering from various diseases [141, 142], whilst the exact cleavage form(s) accumulating in most of these cases remains to be determined. sCD95L was initially believed to be a competitor of its membrane-bound counterpart (mCD95L) in the interaction with CD95 and the consequent induction of the apoptotic signal. It is only in the last decade that it has been reported that not only sCD95L failed in the induction of programmed cell death [143, 144], but that its interaction with CD95 led to the induction of a different type of signal, including engagement of ERK, NF-KB, and PI3K/Akt [145, 146]. Gene-targeted mice selectively lacking either metalloprotease-dependent soluble CD95L (sCD95L) or membrane-bound CD95L (mCD95L) were generated [147]. Mice lacking sCD95L appeared normal and their T cells were able to kill target cells, whereas T cells lacking mCD95L could not kill cells through CD95 activation. Furthermore, mice lacking mCD95L displayed SLE-like symptoms and histiocytic sarcoma. Of note, one group has described that the stimulation of CD95 with sCD95L can induce a calcium-dependent process that leads to the activation of a c-yes/PLCγ1/PI3K/Akt pathway promoting Triple Negative Breast Cancer (TNBC) cell migration [141] (Fig. 3).

mCD95L has also been shown to activate the PI3K/Akt pathway. There appears to be a crosstalk between the two signaling paths PI3K and NF-κB under mCD95L stimulation. Indeed in mutant PIK3CA (PI3K alpha catalytic subunit), but not WT PIK3CA-expressing Hct116 cells, TRAIL, and CD95L stimulated NF-κB activation [148]. It is now clear that caspase-8 not only mediates the cell death signal initiated by CD95L, but also contributes to the induction of apoptosis-independent pathways, such as cell migration and adhesion. Caspase-8 was found to be a substrate of Src kinase c-yes [149]. It has been observed that the stimulation of motility through the EGF, first activates c-yes, and then triggers the phosphorylation of caspase-8 on Tyrosine-380 in the linker region between the two subunits (i.e., p18 and p10) of the procaspase-8 converting it from a pro-apoptotic factor to a cell motility factor. The Y380 phosphorylation prevented downstream activation of the caspase cascade proving a valuable path to explore for sensitization of CD95-resistant tumors to extrinsic apoptotic stimuli [150]. The catalytic domain of caspase-8 is in fact not required for the induction of the migration signal. Once phosphorylated, caspase-8 interacts with the p85 alpha subunit of PI3K (Fig. 3) [151].

In a mouse cell model of glioblastoma (GBM) the c-yes/PI3K-p85 interaction was reported to signal cell invasion via glycogen synthase kinase 3-beta pathway and subsequent expression of matrix metalloproteinases [152]. Blockade of CD95-mediated activity in this cellular model drastically reduced the number of invading cells. In the same context CD95 expression associates with stemness and EMT features and poorer overall survival. CD95-mediated activation of the PI3K p85 also maintained the expression of EMT-related transcripts. The authors therefore suggested that CD95 would be a potential prognostic biomarker in GBM [153].

### Systemic autoimmune diseases

To date, more than 80 diseases in which the etiology is certainly, or most likely, autoimmune have been described [154]. Around the 1960s/70s the distinction was made between systemic autoimmune diseases, with general signs and symptoms and the involvement of multiple organs and systems, and organ-specific autoimmune diseases, where the immuno-pathological damage is localized to an organ and the clinical picture closely linked to the dysfunction of the organ itself. The *self* and *non-self* recognition functions are carried out through an elaborate identification system that involves T and B lymphocytes. The central selection process eliminates the vast majority of auto-reactive lymphocytes at an immature stage during their development, through Bcl-2-interacting mediators of cell death, such as Bim [155]. However, despite the numerous central tolerance mechanisms, many mature B and T lymphocytes, generated in the central lymphoid organs, then reach the peripheral lymphoid organs and undergo activation, turning into their self-reactive form [156, 157]. The Bim-dependent apoptotic pathway is required both for the killing of self-reactive immature B and T lymphocytes during their development and for the elimination of auto-reactive mature B and T lymphocytes in peripheral lymphoid organs [158]. However, except in the thymus, most of the TCR-related mature T-cell apoptosis is induced by the extrinsic pathway via membrane DR, and in particular one of the most important elements of this regulation is apoptosis activated by the CD95/CD95L system [159]. The importance of CD95 and CD95L in eliminating activated T cells is underlined by the anomalies observed when mutations in the genes coding for CD95 or CD95L occur. The CD95/CD95L system has a dual role in immune regulation [160–163]. It constitutes one of the mechanisms by which cytotoxic lymphocytes kill the target, but is also involved in the process of turning off the response. Activation of the lymphocytes leads to an increase in CD95L expression and the ability to trigger apoptosis. Recently Heikenwälder’s group reported that blocking CD95L could prevent auto-aggression of hepatocytes by CD8^pos^ T cells in the precancerous context of Non-alcoholic steatohepatitis (NASH). The liver cells coming into contact with aberrantly activated CD8^pos^ T cells die by apoptosis due to contact with the CD95L overexpressed in these reactive T cells [164]. The same process could occur and damage other organs as well. This observation, made on a mouse model, could be useful for the design of future immunotherapies without affecting the antigen-specific T-cell immunity.

Peripheral T-cell CD95-induced apoptosis eliminates over-activated and self-reactive T cells via a mechanism called “Activation-Induced Cell Death” (AICD) [165]. T-cell activation is also associated with CD95L expression at the cell surface, thus representing a specific aspect of the immune system. AICD is induced through the interaction between CD95 and CD95L, both expressed on activated T cells surface [166]. Similarly to T cells, it has been reported that not only B cells are capable of expressing CD95L but that the level of CD95L expression correlates with the level of activity of B cells, thus making them capable of killing CD95 expressing cells [167]. Consequently, the abnormal activation of these CD95L-expressing B cells is implicated in the immune modulation of various diseases and thus constitutes a therapeutic target [168, 169]. The constitutive expression of CD95L in some “immunologically privileged” tissues (e.g., the Sertoli cells, the testes, or the anterior chamber of the eye), has suggested that CD95L plays also an important role in reducing the activity of immune cells in these tissues. Several studies exploring mutations in the genes encoding CD95 and CD95L have allowed to better understand the pathogenesis of autoimmune diseases, such as ALPS (Autoimmune LymphoProliferative Syndromes) or SLE (Systemic Lupus Erythematous).

#### ALPS: Autoimmune lymphoproliferative syndromes

Some *FAS* gene mutations impair the function of the molecule, leading either to a reduced expression on the membrane, or to the impairment of the ability to transmit the apoptotic signals [170]. The defective shutdown of the immune response resulting from the defective function of CD95 can be the cause of both the progressive accumulation of lymphocytes in the peripheral lymphatic organs and the development of autoimmune reactions [171]. The most common trigger of ALPS is due to autosomal dominant mutations of the *FAS* gene [172, 173], and, less frequently, of *FASLG*, the gene encoding the CD95 ligand [174]. Much less common forms of autoimmune lymphoproliferation are due to mutations in another factor in the T-cell apoptosis pathway, caspase-10. Controversial studies have been carried out in this regard as several heterozygous *CASP10* variants have been identified along with variants known to be polymorphic. It has recently been observed that said *CASP10* mutations are capable of impaired apoptosis [175]. In ALPS patients lacking germline mutations in *FAS*, some dominant somatic mutations in the DR and notably in the Death Domain were found. These somatic mutations were identified as missense variants likely to change the normal structure and impact the oligomerization and functionality of CD95 [176]. Of note, a large study in a cohort of 100 ALPS patients with CD95 DD mutations reported that the risk of non-Hodgkin and Hodgkin lymphomas, respectively, was 14 and 51 times greater than expected [177]. Collectively, all diseases associated with abnormal lymphocyte apoptosis, lymphoproliferation, and autoimmunity, are named Autoimmune Proliferative Syndromes. The syndromes can be classified according to the mutated gene(s) responsible for the defect and they are usually characterized by lymphadenomegaly and hepatosplenomegaly associated with autoimmune manifestations, mainly of the hematological type, such as hemolytic anemia, thrombocytopenia, and neutropenia, as well as the presence of cell-type-specific autoantibodies [178–180]. Furthermore, the accumulation of a minority population of self-reactive CD3^pos^ TCRα^pos^ CD4^neg^ CD8^neg^ T cells called double negative (DNT) has been reported in the early 1990s as a major feature of ALPS. Despite their similarity to normal differentiated T cells, DNTs are remarkably proliferative, particularly in the paracortical region [181]. Last year Kimberly Gilmour’s group carried out a study on 215 patients with clinical evidence of ALPS, intending to define the most useful and predictable biomarkers for a better ALPS diagnosis. Among the several subgroups of patients, levels of different biomarkers, including DNTs and sCD95L, were observed significantly higher in the ALPS-FAS patients than in the “unknown ALPS” (ALPS-U), cases for which the genetic determinant is not identified. They developed a diagnostic protocol for the potential identification of patients with presymptomatic or mild disease. The combination of such biomarkers could be useful in the process of confirming or excluding the ALPS diagnosis [182]. Today the diagnosis requires performing a cell apoptosis test and molecular type analysis, and the choice of therapy is guided by the severity and nature of the symptoms, but generally it is based on the intake of immunosuppressants such as rituximab. The increased sCD95L serum levels are now part of the new diagnostic criteria procedure for an ALPS [183, 184]; these high levels have indeed been associated with the pathology without their pathophysiological role being elucidated [185]. Curiously, some groups observed a change in sCD95L levels in correlation with aging, and age-related conditions and/or diseases with an increase in molecular signals due to aging oxidative stress [186]. Furthermore, oxidative stress seems to promote CD95L cleavage through activation of MMPs, and more interestingly this MMP activation seems to increase as a function of aging [187].

#### SLE: Systemic lupus erythematous

In both humans and mice, mutations in the genes coding for CD95 or CD95L are also strongly associated with certain forms of lupus disease. The defects in apoptosis described in autoimmunity and lymphoproliferative syndromes correspond to the human equivalent of the *MRL/lpr* mouse model *(Murphy Roths Large/lymphoproliferation)*, deeply investigated as a murine SLE-like model [188, 189]. This model was generated following the identification of an autosomal recessive modification on chromosome 19 [190]. The mentioned mutation was found on the gene encoding CD95 protein. Similar to this model, a second model was designed and generated after the discovery of a second autosomal recessive mutation, on chromosome 1, corresponding to the gene coding for CD95L [191]. The latter model took the name of *MRL*/*gld* for generalized lymphoproliferative disease. In addition to those two mouse models, a wider selection of mouse models is available to sift genetic and cellular aspects of SLE [192, 193]. Since the etiology of SLE is multifactorial and multigenetic, some of these models, such as those mentioned above, derive from spontaneous genetic factors, while others assume a SLE-like phenotype after exposure to certain chemicals such as intraperitoneal injections of pristane (2, 6, 10, 14 tetramethylpentadecane) [194], or overexpression of cytokines (ie IL-6, IL-12, INF-I) [195–197]. Others, similarly to induced graft-versus-host disease models, develop a lupus-like syndrome following donor cell injection [198]. Despite their numerous limitations, over the years these SLE-like mouse models have been widely used to screen numerous potential therapies, pointing out their importance in the study of this disease and in the therapeutic advancement in this field [199]. Systemic lupus erythematosus is a rare systemic autoimmune disease, more severe in women, especially of childbearing age. Very recently Lars Rönnblom’s group has observed that there is a correlation between the cumulative genetic risk and survival, organ damage, renal dysfunctions, in patients affected by SLE, introducing Genetic Risks Score (GRS) as a potential tool for predicting outcomes in patients with SLE [200]. The term “systemic” means that the disease affects several organs. Genetically speaking, germline heterozygous mutations in the *FAS* gene have been observed in pediatric cases with ALPS-FAS. These children develop symptoms similar to those of systemic lupus erythematosus disease [201]. According to Neven’s report, *FAS* mutations were located within the intracellular domain of CD95. On the other hand, germline mutations in the *FASLG* gene seem to be involved only in a minority of patients with SLE. This does not exclude the possible role of somatic mutations in the *FASLG* gene in some of the self-reactive clones that contribute to the expression of the disease [202]. High levels of sCD95L have also been detected in the serum of SLE patients, compared to those present in the serum of healthy donors [142]. This observation seems to suggest that high levels of sCD95L may be related to the aggravation of the disease, which constitute a new opening for the study of new therapeutic strategies. Indeed, to date, there are unfortunately no targeted therapies against SLE disease. The diagnosis of this heterogeneous disease is not always simple, as in the early stages the symptoms can simulate other pathologies. For instance, the first “red flags” are given by skin and joint symptoms, both of which can be traced back to multiple pathologies. Less commonly, various infections, as well as pathological conditions such as mixed connective tissue disease (MCTD) or sarcoidosis, can mimic the symptoms of lupus. As a general rule, the first test to be performed is the fluorescence analysis for the detection of antinuclear antibodies (ANA), commonly called autoantibodies. Indeed, 98% of patients with systemic lupus have a positive immunofluorescent ANA test. Several blood and kidney involvement tests are later performed to support the latter. Patients with SLE frequently develop haematopathological and nephropathological conditions, such as leukopenia, thrombocytopenia, hemolytic anemia and active nephritis [203, 204]. The treatment of lupus is standardized and involves corticosteroids, immunosuppressants, and non-steroidal anti-inflammatory drugs in addition to hydroxychloroquine for mild disease [205, 206]. As for new therapeutic options, a large number of drugs, mainly monoclonal antibodies (mAbs), have been evaluated and tested with rather disappointing results. The main objective is to reduce the doses of corticosteroids and immunosuppressants used, as a chronic administration of these drugs causes complications such as infections or secondary osteoporosis [207, 208]. To date, Belimumab (anti-B-cell activating factor) is the only biotherapeutic approved for the treatment of the non-renal form of SLE [209]. The use of Belimumab as an addition to standard therapies seems to improve the quality of life of patients suffering from this disease, but the goal to replace “conventional” drugs remains to be demonstrated. The study conducted on the use of other monoclonal antibodies, for instance Rituximab (anti-CD20) and Anifrolumab (anti-type I interferon receptor), for the treatment of this pathological condition is still ongoing.

#### Organ-specific autoimmunity

In contrast to systemic autoimmune diseases, organ-specific autoimmunity is characterized by a cell-mediated attack against a specific type of cell in a given target organ, thus causing tissue damage. Some examples of such clinical conditions are insulin-dependent diabetes mellitus, ulcerative colitis (UC), multiple sclerosis (MS), or Sjögren’s syndrome (SS), all conditions to which excessive CD95-mediated apoptosis can contribute [210, 211]. As previously stated, the CD95/CD95L complex plays a central role in controlling immune reactions via AICD. This process is crucial in regulating the autoantigen-dependent primary T-cell response. Therefore, CD95L-mediated AICD dysregulation could be implicated in the acceleration process of organ-specific autoimmune lesions. Furthermore, sCD95L secretion is generally increased in effector cells upon specific activation with organ-specific autoantigen [212]. sCD95L could thus act as an inhibitor of CD95-mediated AICD in these contexts, promoting effector T-cell proliferation and tissue lesions, as demonstrated for antoantigen-reactive CD4 T cells in SS mouse models [212]. Over the years, the numerous studies carried out on the role of the soluble form of CD95L in the context of organ-specific autoimmune diseases, have led to conflicting results. It seems that the role of soluble CD95L varies according to the type of disorder and the mouse model used. In 2019 a group showed on non-obese diabetes mice (NOD) lacking sCD95L and maintaining mCD95L and immune homeostasis that sCD95L does not markedly affect islet inflammation, hence the pathogenesis of autoimmune diabetes, but more interestingly that sCD95L deficiency does not alter immune homeostasis in NOD mice [213].

## Currently used cancer therapies

Since the discovery of CD95 [214–217], it has been thought possible to exploit the physiological importance of CD95/CD95L to develop new powerful chemotherapeutic agents. However, it was quite soon discovered that systemic administration of CD95 agonists resulted in severe toxicity [218]. It was observed that these agents induced massive apoptosis of hepatocytes resulting in a form of fulminant hepatitis, lethal to the treated animals [219, 220]. Over the past two decades, controversies over the different implications assumed by the CD95/CD95L system in various diseases such as cancer, autoimmune diseases and inflammatory diseases have made it difficult to identify targeted therapies. Several studies have developed interesting approaches to strengthen the apoptotic function of CD95 and limit the side effects deriving from the non-specificity of the previously developed molecules. Some of these studies will be described in this review. Unfortunately, very few of these approaches have reached clinical trials (Table 1: *Breakdown of the patents targeting Death Receptors and/or their ligand*).

**Table 1:** Table listing the 127 patents published in the last 25 years concerning CD95 or its cognate ligand CD95L or the entire CD95/CD95L interaction system.

N°	Publication Id	Publication date	Country	Disease	Approach	Title	Inventors	Ref.
1	WO2003079750	2003.10.02	WO	Autoimmune diseases/inflammatory diseases/cancer	Antibodies	Antagonistic anti-hfas ligand human antibodies and fragments thereof	LANCASTER Joanne Sloan	[350]
2	US20200102397/WO2017051002	2020.04.02/2017.03.30	WO	Cancer	Antibodies	Anti-cd95l antibody	GIEFFERS Christian, HILL Oliver, THIEMANN Meinolf et al.	[221]
3	WO2015165973	2017.03.08	WO	Cancer	Antibodies	Diagnostic anti-cd95l antibody	FRICKE, Harald GIEFFERS, Christian SYKORA, Jaromir	[221]
4	WO2008080623	2016.04.28	WO	Cancer	Antibodies	Neutralization of cd95 activity blocks invasion of glioblastoma cells in vivo	MARTIN-VILLALBA Ana, KLEBER Susanne, WIESTLER Benedikt et al.	[325]
5	US20150274833/EP2920210/WO2014076292	2015.10.01/2015.09.23/2014.05.22	WO	Cancer	Antibodies	Recombinant bispecific antibody binding to cd20 and cd95	HERRMANN Andreas, GROSSE-HOVEST Ludger	[271, 272, 351]
6	EP2717911/WO2012168259	2014.04.16/2012.12.13	WO	Cancer	Antibodies	Protein tyrosine phosphatase, non-receptor type 11 (ptpn11) and triple-negative breast cancer	BENTIRES-ALJ Mohamed, ACETO Nicola, STADLER Michael	[352]
7	US20060083738/EP1506237/WO2003097698	2006.04.20/2005.02.16/2003.11.27	WO	Cancer	Antibodies	Treatment of cancer by the use of anti fas antibody	JOHNSTON Patrick Gerard, LONGLEY Daniel	[269, 353–355]
8	WO2010066914	2011.10.19	WO	Others	Antibodies	Remedies for pemphigus containing anti fas-ligand antibodies	PINCELLI Carlo, MARCONI Alessandra	[356]
9	US20030082180/WO2001041803	2003.05.01/2001.06.14	WO	Others	Antibodies	Combination of compounds that inhibit the biological effects of tnf-α and cd95l in a medicament	KRAMMER Peter, MARTIN-VILLALBA Ana	[357]
10	US20110300113/EP2064235/WO2008034608	2011.12.08/2009.06.03/2008.03.27	WO	Others	Antibodies/cells	The death receptor cd95 controls neurogenesis of adult neural stem cells in vivo and in vitro	MARTIN-VILLALBA Ana, CORSINI Nina, LETELLIER Elisabeth et al.	[358]
11	WO2010006772	2011.08.04	WO	Inflammatory diseases	Antibodies/fusion proteins	Use of cd95 inhibitors for the treatment of inflammatory disorders	MARTIN-VILLALBA Ana, LETELLIER Elisabeth, SANCHO-MARTINEZ Ignacio	[359]
12	US20170166648/EP3150224/WO2014013036	2017.06.15/2017.04.05/2014.01.23	WO	Others	Antibodies/fusion proteins	Inhibitors of the cd95 signaling pathway for treatment of mds	FRICKE Harald, FONTENAY Michaela, KUNZ Claudia	[348, 349]
13	US20060234968/WO2004071528	2006.10.19/2004.08.26	WO	Others	Antibodies/fusion proteins	Inhibition of the cd95 ligand/receptor system for the treatment of neurological disorders and injuries	MARTIN-VILLALBA Ana, KRAMMER Peter, DEMJEN Deana	[360]
14	US20030170244	2003.09.11	US	Cancer/others	Antibodies/fusion proteins	Inhibition of fas signaling	PLUENNEKE John D, CONNOR Timothy	[361]
15	US6846637/WO1999065935	2005.01.25/1999.12.23	WO	Cancer/autoimmune diseases/others	Antibodies/polypeptides	Fas peptides and antibodies for modulating apoptosis	CHIODI Francesca	[362]
16	WO2016170027	2016.10.27	WO	Inflammatory diseases	Antibodies/polypeptides	Methods and pharmaceutical compositions for the treatment of th17 mediated diseases	LEGEMBRE Patrick, BLANCO Patrick, FLYNN Robin	[101]
17	US20120294856/EP2502069/WO2011058175	2012.11.22/2012.09.26/2011.05.19	WO	Cancer	Antobodies/enzymes/RNA interfering molecules	Compounds inhibiting cd95 signaling for the treatment of pancreatic cancer	MARTIN-VILALBA Ana, HERHAUS Peter, SANCHO-MARTINEZ Ignacio et al.	[363]
18	US20200407728/WO2016069282	2020.12.31/2016.05.06	WO	Autoimmune diseases	Cells	Altering gene expression in modified t cells and uses thereof	ZHAO Yangbing, REN Jiangtao, LIU Xiaojun, JUNE Carl H.	[364]
19	US20150104428/EP2833896/WO2013149211	2015.04.16/2015.02.11/2013.10.03	WO	Autoimmune diseases	Cells	Compositions and treatment methods for mesenchymal stem cell-induced immunoregulation	SHI Songtao, AKIYAMA Kentaro, CHEN Chider	[330, 331]
20	WO2015038665	2015.03.19	WO	Autoimmune diseases/inflammatory diseases	Cells	A composition of stem cells having highly expressed fas ligand	SHI Songtao, LIU Shiyu, CHEN Fa-ming	[365]
21	US20200121719/EP3565888/WO2018129332	2020.04.23/2019.11.13/2018.07.12	WO	Cancer	Cells	Expansion of tumor-infiltrating lymphocytes (tils) with tumor necrosis factor receptor superfamily (tnfrsf) agonists and therapeutic combinations of tils and tnfrsf agonists	LOTZE Michael, T, RITTHIPICHAI Krit	[277, 366, 367]
22	EP3569700/WO2011052545	2019.11.20/2011.05.05	WO	Cancer	Cells	Method for producing antigen-specific b-cell population	KITAMURA Daisuke, NOJIMA Takuya	[368]
23	US20180008670	2018.01.11	US	Cancer	Cells	Chimeric antigen receptor targeting of tumor endothelium	WAGNER Samuel C., ICHIM Thomas E, MINEV Boris	[280]
24	WO2015161276	2015.10.22	WO	Cancer	Cells	Crispr-cas-related methods, compositions and components for cancer immunotherapy	WELSTEAD G. Grant, FRIEDLAND Ari E, MAEDER Morgan L, BUMCROT David A	[369]
25	WO2014039044	2014.13.03	US	Cancer	Cells	Methods of producing t memory stem-cell populations	GATTINONI Luca, LUGLI Enrico, ROEDERER Mario, RESTIFO Nicholas P	[281, 370, 371]
26	US20040131599/WO2002072798	2004.07.08/2002.09.19	WO	Cancer	Cells	Fas ligand-expressing hematopoietic cells for transplantation	CIVIN Curt, I, DRACHMAN Daniel, WHARTENBY Katherine, PARDOLL Drew M	[372]
27	US20090191167/EP2046351/WO2008014470	2009.07.30/2009.04.15/2008.01.31	WO	Cancer/autoimmune diseases/others	Cells	Adult sertoli cells and uses thereof	WHITE David J	[373]
28	WO2018227286	2018.12.20	WO	Inflammatory diseases/others	Cells	Allograft tolerance without the need for systemic immune suppression	NAGY Andras, HARDING Jeffrey, NAGY Kristina	[374]
29	WO2018130679	2019.11.20	WO	Cancer	Chemicals	Methods and pharmaceutical compositions for reducing cd95- mediated cell motility	LEGEMBRE Patrick, VACHER Pierre, POISSONNIER Amanda, BLANCO Patrick	[101]
30	US20190084987	2019.03.21	US	Cancer	Chemicals	Small molecule histone methyltransferase suv39h1 inhibitor and uses thereof	LU Chunwan, LEBEDYEVA Iryna, LIU Kebin	[301]
31	US20150343024/EP2931375/WO2014090224	2015.12.03/2015.10.21/2014.06.19	WO	Cancer	Chemicals	Use of active substance combinations for inducing tumor senescence	RÖCKEN Martin, WIEDER Thomas, HAHN Matthias et al.	[375]
32	WO2008036244	2008.03.27	WO	Cancer	Chemicals	Use of cyclosporin a to sensitize resistant cancer cells to death receptor ligands	REED John C, THOMAS Michael P	[376]
33	WO2019206834	2019.10.31	WO	Cancer/autoimmune dieases	Chemicals	Compounds and pharmaceutical compositions for reducing cd95-mediated cell motility	VACHER Pierre, LEGEMBRE Patrick, JEAN Mickael et al.	[142]
34	WO2002047728	2002.06.20	WO	Others	Chemicals	Treatment of posterior capsule opacification	ALLAN Bruce Duncan Samuel	[377]
35	WO2019141862	2019.07.25	WO	Inflammatory diseases	Chemicals/antobodies/fusion proteins/nucleotide complexes	Combination therapeutics	WALCZAK Henning, TARABORRELLI Lucia, PELTZER Nieves	[321]
36	US20090142369	2009.06.04	US	Others	Cosmetic composition	Method for preventing skin-cellular injury by using green algae extract and cosmetic composition containing green algae extract	SHIH Meng-Han, SHIH Mei-Fen	[378]
37	US20080118466	2008.05.22	US	Autoimmune diseases	Drug delivery system	Treatment of rheumatoid arthritis with soluble fas-ligand cross-linkers	HUREZ Vincent Jacques, MICHELSON Seth G, SHODA Lisl Katharine et al.	[379, 380]
38	EP0930890/WO1998017305	1999.07.28/1998.04.30	WO	Autoimmune diseases	Drug delivery system	Use of fasl or fasl transfected cd4?+ /fasl?- /th1-cell lines for the treatment of th1/th2 diseases	HAHNE Michael, TSCHOPP Juerg, DA CONCEICAO-Silva Fatima, SCHROETER Michael	[381]
39	US20200046780/EP3592392	2020.02.13/2020.01.15	US/EP	Autoimmune diseases/others	Drug delivery system	Fasl-engineered biomaterials with immunomodulatory function	SHIRWAN Haval, GARCIA Andres J, YOLCU Esma S et al.	[136, 382]
40	US20050214311/EP1478390/WO2003070271	2005.09.29/2004.11.24/2003.08.28	WO	Cancer	Drug delivery system	Novel complexes for inducing an immune response	SCREATON Gavin R, SIMON Katharina A, GALLIMORE Awen M	[383]
41	US20200108117/WO2019246130	2020.04.09/2019.12.26	US	Cancer/others	Drug delivery system	Drug delivery systems comprising an intraocular pressure lowering agent, a neurotrophic agent, a c-type natriuretic peptide, a natriuretic peptide receptor-b, an apoptosis signaling fragment inhibitor or a fas-ligand inhibitor for treating glaucoma or ocular hypertension	SCHIFFMAN Rhett M, SCHEIBLER Lukas	[384]
42	US20200246432/WO2019040372	2020.08.06/2019.02.28	WO	Others	Drug delivery system	Nitric oxide- and fas ligand- eluting compositions and devices and methods of treatment using same	KURAL M Hamdi, GUI Liqiong, NIKLASON L Elizabeth, SALTZMAN William Mark	[385]
43	WO2019246141	2019.12.26	WO	Others	Drug delivery system	Drug delivery systems comprising a neurotrophic agent, an apoptosis signaling fragment inhibitor (fas) or fas-ligand (fasl) inhibitor, a tumor necrosis factor-α (tnf-α) or tnf receptor inhibitor, a mitochondrial peptide, an oligonucleotide, a chemokine inhibitor, or a cysteine-aspartic protease	SCHIFFMAN Rhett M, SCHEIBLER Lukas	[384]
44	WO2019246130	2019.12.26	WO	Others	Drug delivery system	Sustained-release drug delivery systems comprising an intraocular pressure lowering agent, a cnp compound, an npr-b compound, a tie-2 agonist, or neurotrophic agent for use for treating glaucoma or ocular hypertension	SCHIFFMAN Rhett M, SCHEIBLER Lukas	[384]
45	US20040096450/WO2000040263	2004.05.20/2000.07.13	WO	Others	Drug delivery system	Methods and compositions for treating diseases associated with increased fas-ligand titers	FRENCH Lars E, VIARD Isabelle, TSCHOPP Jurg	[386]
46	US20190330305/WO2014121085	2019.10.31/2014.08.14	WO	Autoimmune diseases/inflammatory diseases/cancer	Fusion proteins	Pd-l1 and pd-l2-based fusion proteins and uses thereof	TYKOCINSKI Mark L	[387]
47	EP1481687	2004.12.01	EP	Autoimmune diseases/inflammatory diseases/cancer	Fusion proteins	Use of multimeric ligands of the tnf family with reduced toxicity for treating cell proliferative diseases	ROSAT Jean-Pierre	[294]
48	US20070269449/WO2004085478	2007.11.22/2004.10.07	WO	Autoimmune diseases/inflammatory diseases/others	Fusion proteins	Cd95-fc fusion proteins	WALCZAK Henning	[388]
49	EP1214411/WO2001018202	2002.06.19/2001.03.15	WO	Autoimmune diseases/inflammatory diseases/others	Fusion proteins	Flint analog compounds and formulations thereof	ATKINSON Paul Robert, TIAN Yu, WITCHER Derrick Ryan	[389–391]
50	WO2001090382	2001.11.29	WO	Autoimmune diseases/inflammatory diseases/others	Fusion proteins	Fas ligand-fused proteins	TOUMA Jyunko	[392]
51	US20180318394/EP3313429/WO2016205714	2018.11.08/2018.05.02/2016.12.22	WO	Autoimmune diseases/others	Fusion proteins	Immunomodulation for the long-term prevention and treatment of autoimmune diseases and foreign tissue rejection	SHIRWAN Haval	[382, 393, 394]
52	US20110081369/EP1250055	2011.04.07/2002.10.23	US/EP	Autoimmune diseases/others	Fusion proteins	Methods of immune modulation with death receptor-induced apoptosis	SHIRWAN Haval	[382, 393, 394]
53	US20110003385/EP0804561	2011.01.06/1997.11.05	US/EP	Autoimmune diseases/others	Fusion proteins	Regulated transcription of targeted genes and other biological events	CRABTREE Gerald R, SCHREIBER Stuart L, SPENCER David M	[395]
54	US20090239240/EP2039768	2009.09.24/2009.03.25	US	Autoimmune diseases/others	Fusion proteins	Mutant forms of fas ligand and uses thereof	KETING Chu	[266]
55	US20180148512/WO2014121093	2018.05.31/2014.08.07	WO	Cancer	Fusion proteins	Fusion proteins that facilitate cancer cell destruction	TYKOCINSKI Mark L	[289]
56	WO2015197874	2017.05.03	WO	Cancer	Fusion proteins	Combination of cd95/cd95l inhibition and cancer immunotherapy	KUNZ Claudia, FRICKE Harald, HÖGER Thomas, GAMER Juergen	[266, 396–398]
57	US20150297745/EP2897642/WO2014045022	2015.10.22/2015.07.29/2014.03.27	WO	Cancer	Fusion proteins	Agents and methods	COBBOLD Mark, MILLAR David	[399]
58	WO2012170072	2014.04.17	WO	Cancer	Fusion proteins	Engineered antibody-tnfsf member ligand fusion molecules	GREWAL Iqbal, KHARE Sanjay D, GRESSER Michael, SYED Rashid	
59	US20120177575/EP2456468/WO2011010156	2012.07.12/2012.05.30/2011.01.27	WO	Cancer	Fusion proteins	Fas (apo-1,cd95) targeted platforms for intracellular drug delivery	ATEH Davidson D, MARTIN Joanne E	[400]
60	US20110008842	2011.01.13	US	Cancer	Fusion proteins	Chimeric nucleic acids encoding polypeptides comprising cd70 and fas-ligand domains	PRUSSAK Charles E, KIPPS Thomas J, CANTWELL Mark J	[401]
61	EP3406630/US20180186856/WO2014013037	2018.11.28/2018.02.22/2014.01.23	WO	Cancer/autoimmune diseases/inflammatory diseases/others	Fusion proteins	Nucleic acids encoding artificial signal peptides and methods of production thereof	HILL Oliver, GIEFFERS Christian, THIEMANN Meinolf	[324, 325, 346, 348, 402]
62	WO2014013039	2014.01.23	WO	Cancer/autoimmune diseases/inflammatory diseases/others	Fusion proteins	Composition comprising a mixture of cd95-fc isoforms	HILL Oliver, GIEFFERS Christian, THIEMANN Meinolf	[324, 325, 402]
63	WO2013060864	2014.09.03	WO	Cancer/autoimmune diseases/others	Fusion proteins	Chimeric molecule involving oligomerized fasl extracellular domain	TAUPIN Jean-Luc, DABURON Sophie, MOREAU Jean-François, CAPONE Myriam	[292, 293]
64	WO2008025516	2011.02.03	WO	Cancer/inflammatory diseases/others	Fusion proteins	Cd95l or trail fusion proteins	HILL Oliver, GIEFFERS Christian, THIEMANN Meinolf	[324, 325, 402]
65	US20190016782/WO2014106839	2019.01.17/2014.07.10	WO	Cancer/others	Fusion proteins	Stable form of signal converting protein fusion proteins, and methods of use and preparation thereof	DRANITZKI ELHALEL Michal, SHANI Noam	[266]
66	EP2042509/US20070154905 /WO1997003998	2009.04.01/2007.07.05/1997.02.06	WO	Cancer/others	Fusion proteins	Modulators of the function of fas receptors and other proteins	WALLACH David, BOLDIN Mark, GONCHAROV Tanya, GOLSTEV Yury V	[403]
67	WO2007022273	2007.02.22	WO	Cancer/others	Fusion proteins	Vegf-activated fas ligands	QUINN Timothy P	[404, 405]
68	US20040147447/WO1999066039	2004.07.29/1999.12.23	WO	Cancer/others	Fusion proteins	Tnfr-like protein with death domain	LU Jian J, GOMES Bruce C, FIELES William E	
69	US20110171212	2011.07.14	US	Inflammatory diseases	Fusion proteins	Methods and compositions for preventing radiation-induced pneumonitis	BELKA Claus HERBST Jörg	[406]
70	US20040176279/WO2002060949	2002.08.08	WO	Inflammatory diseases/others	Fusion proteins	Glycoforms a fas-ligand inhibitory protein analog	JENKINS Nigel, WITCHER Derrick R WROBLEWSKI Victor J	[407]
71	US20160340409/EP2621514/WO2012042480	2016.11.24/2013.08.07/2012.04.05	WO	Others	Fusion proteins	Compositions and methods for treatment of hematological malignancies	DRANITZKI ELHALEL Michal	[266]
72	US20040018170	2004.01.29	US	Others	Fusion proteins	Fas ligand-avidin/streptavidin fusion proteins	SHIRWAN Haval	[382, 393, 394]
73	EP1097226/WO2000003023	2001.05.09/2000.01.20	WO	Cancer/autoimmune diseases/others	Fusion proteins	Usurpin, a mammalian ded-caspase homologue that precludes caspase-8 recruitment and activation by the cd95 (fas, apo-1) receptor complex	NICHOLSON Donald, W, RASPER Dita M, XANTHOUDAKIS Steve, ROY Sophie	[408]
74	EP3337509/US20170051352	2018.06.27/2017.02.23	US/EP	Autoimmune diseases	Method	Methods of treating autoimmune conditions in patients with genetic variations in dcr3 or in a dcr3 network gene	HAKONARSON Hakon, KAO Charlly, CARDINALE Christopher et al.	[409]
75	US20160194714	2016.07.07	US	Autoimmune diseases	Method	Biomarkers for predicting relapse in multiple sclerosis	RUS Horea TEGLA Cosmin	[306–309]
76	US20160208332	2016.04.07	US	Autoimmune diseases	Method	Diagnosis and prognosis of multiple sclerosis	RUS Horea CUDRICI Cornelia TEGLA Cosmin	[306–309]
77	US20100285600/EP1891233/WO2006116602	2010.11.11/2008.02.27/2006.11.02	WO	Autoimmune diseases	Method	Markers associated with the therapeutic efficacy of glatiramer acetate	LANCET Doron BECKMANN Jacques AVIDAN Nili et al.	[410]
78	US20090074870/WO2002002751	2009.03.19/2002.01.10	WO	Autoimmune diseases/others	Method	Alteration of cell membrane with fasl	SHIRWAN Haval	[411]
79	WO2015107105	2016.11.23	WO	Cancer	Method	Method of predicting the responsiveness of a cancer disease to treatment on the basis of dna methylation	FRICKE Harald	[297–299]
80	WO2015104284	2016.11.16	WO	Cancer	Method	Methods and pharmaceutical compositions for preventing or reducing metastatic dissemination	LEGEMBRE Patrick, SEGUI Bruno, LEVADE Thierry, MICHEAU Olivier	[296, 412]
81	WO2014118317	2015.12.31	WO	Cancer	Method	Methods for predicting and preventing metastasis in triple-negative breast cancers	LEGEMBRE Patrick, MALLETER Marine, TAUZIN Sébastien et al.	[141]
82	US20150098924/WO2006037762	2015.04.09/2006.04.13	WO	Cancer	Method	Method for ex-vivo purging in autologous transplantation	DUPUIS Marc, GREANEY Peter, DUCHOSAL Michel	[413]
83	EP1668360/US20050069963	2006.06.14/2005.03.31	US/EP	Cancer	Method	Multifactorial assay for cancer detection	LOKSHIN Anna E. GORELIK Elieser	[414]
84	US20050158807/WO2003056340	2005.07.21/2003.07.10	WO	Cancer	Method	Fadd proteins, phosphorylated p38-mapk and fasl as tumor markers	CHIOCCHIA Gilles, TOURNEUR Lea, FEUNTEUN Jean et al.	[415]
85	WO2005053739	2005.06.16	WO	Cancer	Method	Combination therapy	JOHNSTON Patrick G, LONGLEY Daniel	[416]
86	EP1127075/WO2000027883	2001.08.29/2000.05.18	WO	Cancer	Method	A method of treating tumors using fas-induced apoptosis	DONG Jian-Yun NORRIS James S	[417]
87	WO2001048238	2001.07.05	WO	Cancer	Method	Chemotherapeutant screening method	KRAMMER Peter, EICHHORST Sören, LI-WEBER Min, MÜLLER-SCHILLING Martina	[418]
88	WO1999003998	1999.01.28	WO	Cancer	Method	Methods and compositions for tumor reduction	NABEL Gary J	[419]
89	US6153385/WO1997020067	2000.11.28/1997.06.05	WO	Cancer/autoimmune diseases/others	Method	Process for detecting the expression of cd95 ligand in cells	DEBATIN Klaus-Michael HERR Ingrid	[420]
90	EP0876503/WO1997020064	1998.11.11/1997.06.05	WO	Cancer/autoimmune diseases/others	Method	Process for assessing the activity of drugs	DEBATIN Klaus-Michael, FRIESEN Claudia, KRAMMER Peter, HERR Ingrid	[420]
91	EP0689600/WO1994020625	1996.01.03/1994.09.15	WO	Cancer/others	Method	Process to induce the death of tumor cells	WONG Grace H W	[421]
92	WO1999003999	1999.01.28	WO	Inflammatory diseases	Method	Methods and compositions for inhibiting the pro-inflammatory response	NABEL Gary J, CHEN Jian-Jun	[422]
93	US20020127233	2002.09.12	US	Inflammatory diseases/cancer/others	Method	Method for inhibiting inflammation in immune privileged sites using fas-ligand fragments	ZHU Bing, CYNADER Max S, PATY Donald W, LUO Liqing	[423]
94	US20180369380	2018.12.27	US	Others	Method	Methods and compositions for treating conditions of the eye	GRAGOUDAS Evangelos S, POULAKI Vasiliki, MILLER Joan W	[424]
95	US20140045198/EP2678688 /WO2012113760	2014.02.13/2014.01.01/2012.08.30	WO	Others	Method	Method of predicting the evolution of a patient suffering of a neurovascular disease	MONTANER VILLALONGA Joan, ROSELL NOVEL Anna, NAVARRO SOBRINO Miriam	[357, 425]
96	US20110294690/EP2338058/WO2010031821	2011.12.01/2011.06.29/2010.03.25	WO	Others	Method	Differential diagnostic biomarkers of stroke mimicking conditions and methods of use thereof	MONTANER VILALLONGA Joan	[357, 425]
97	US20060241150/WO2005000405	2006.10.26/2005.01.06	WO	Others	Method	P38 kinase inhibitor compositions and methods of use	WEINER David B, MUTHUMANI Karuppiah	[426]
98	WO2006077232	2006.07.27	WO	Others	Method	Multimeric soluble fas-ligand for eliminating alloreactive t lymphocyte in allogenic harmatopoietic stem-cell transplantation transplantation	DUPUIS Marc, DEMOTZ Stéphane, GREANEY Peter et al.	[413, 427]
99	US20050129684	2005.06.16	US	Others	Method	Methods for preserving the viability of photoreceptor cells by anti-fas-ligand/anti-fas-receptor antibodies	ZACKS David, MILLER Joan W	[428]
100	US20030224403	2003.12.04	US	Others	Method	Lethal toxin cytopathogenicity and novel approaches to anthrax treatment	POPOV Serguei G, CARRON Edith G, CARDWELL Jennifer	[429]
101	US6524821/WO2000007618	2003.02.25/2000.02.17	WO	Others	Method	Anti-apoptotic compositions comprising the r1 subunit of herpes simplex virus ribonucleotide reductase or its n-terminal portion; and uses thereof	LANGELIER Yves, MASSIE Bernard	[430]
102	US6485929/WO1999036091	2002.11.26/2000.07.22	WO	Others	Method	Method for inhibiting cd95-independent apoptosis in aids	KRAMME, Peter H, BERNDT Christina	[431]
103	WO2015189236	2015.12.17	WO	Cancer	Method	Methods and pharmaceutical compositions for reducing cd95-mediated cell motility	LEGEMBRE Patrick, COUNILLON Laurent, LAGADIC-GOSSMANN Dominique	[295]
104	US2003011865/WO2000059538	2002.06.06/2000.10.12	WO	Autoimmune diseases/others	Nucleotide complexe	Antigen-specific induction of peripheral immune tolerance	AUGUST Thomas J. LEONG Kam W. GEORGANTAS Robert	[432]
105	WO2001051503	2001.07.19	WO	Cancer	Nucleotide complexe	Polynucleotides for inhibiting metastasis and tumor cell growth	BARBERA-GUILLEM Emilio	[433]
106	US20020042064/EP1121438/WO2000023583	2002.04.11/2001.08.08/2000.04.27	WO	Cancer/autoimmune diseases/others	Nucleotide complexe	P53 binding areas	KRAMMER Peter, MÜLLER-SCHILLING Martina, OREN Moshe	[251]
107	US20040033979/EP1176965	2004.02.19/2002.02.06	US/EP	Cancer/autoimmune diseases/inflammatory diseases	Nucleotide complexe	Antisense modulation of fas mediated signaling	DEAN Nicholas M, MARCUSSON Eric G, WYATT Jacqueline, ZHANG Hong	[434]
108	US20030119776/EP1313853	2003.06.26/2003.05.28	US/EP	Cancer/autoimmune diseases/others	Nucleotide complexe	Modulation of fas and fasl expression	PHILLIPS Nigel C, FILION Mario C	[435]
109	US20070190607/WO2001058953	2007.08.16/2001.08.16	WO	Autoimmune diseases	Polypeptides	Inhibitors of pre-ligand assembly doman and function of the tumor necrosis factor receptor family	LENARDO Michael J, CHAN Francis Ka-Ming, SIEGEL Richard M	[36]
110	US7097972/WO1996025501	2006.08.29/1996.08.22	WO	Autoimmune diseases/inflammatory diseases/cancer	Polypeptides	Method and composition for regulating apoptosis	DIXIT,Vishva M	[436]
111	US20120245081	2012.09.27	US	Autoimmune diseases/inflammatory diseases/others	Polypeptides	Fas peptide mimetics and uses thereof	GREENE Mark I, MURALI Ramachandran, HASEGAWA Akihiro	[437]
112	US20070184522/EP1737483/WO2005117940	2007.08.09/2007.01.03/2005.12.15	WO	Autoimmune diseases/others	Polypeptides	Cell death modulation via antagonists of fasl and fas activation	ZARNEGAR Abdolreza, DEFRANCES Marie C, ZOU Chun-Bin	[438]
113	US6451759/WO1999036079	2002.09.17/1999.07.22	WO	Autoimmune diseases/others	Polypeptides	Noncleavable fas ligand	KANG, Sang-Mo BRAAT, Dries BAEKKESKOV, Steinunn STOCK, Peter, G.	[439]
114	WO2020132465	2020.06.25	WO	Cancer	Polypeptides	Methods and compositions related to therapeutic peptides for cancer therapy	BECKER Lev, CUI Chang	[286]
115	EP2102234/WO2008067305	2009.09.23/2008.05.06	EP	Cancer	Polypeptides	Polypeptides comprising intracytoplasmic death domain and nkg2d ligand domain	WAGNER Thomas E, WEI,Yanzhang	[440]
116	US20190085050/WO2015158810	2019.03.21/2015.10.22	WO	Cancer/autoimmune diseases/inflammatory diseases	Polypeptides	Polypeptides and uses thereof for reducing cd95-mediated cell motility	LEGEMBRE Patrick, VACHER Pierre, SANSEAU Doriane et al.	[146]
117	EP1225908/WO2001028582	2002.07.31/2001.04.26	WO	Cancer/inflammatory diseases/others	Polypeptides	Therapeutic applications of flint polypeptides	BUMOL Thomas F COHEN Fredric J	
118	US20100041596/WO2007002633	2010.02.18/2007.01.04	WO	Inflammatory diseases	Polypeptides	Amelioration of inflammatory arthritis by targeting the pre-ligand assembly domain (plad) of tumor necrosis factor receptors	LENARDO Michael, DENG Guo-Min, CHAN Francis Ka-Ming, ZHENG Lixen	[441]
119	US20100041596/WO2009027350	2017.08.31/2009.03.05	WO	Inflammatory diseases/others	Polypeptides	Use of sco-spondin peptides for inhibiting or preventing neuronal apoptosis mediated by cell death receptor ligands	MEINIE, Annie, LALLOUE Fabrice, JAUBERTEAU Marie-Odile	[442]
120	US20130288979/EP2982685 /WO2012066103	2017.11.14/2016.02.10/2012.05.24	WO	Others	Polypeptides	Inhibitors of apoptosis and uses thereof	BARRERE Stéphanie, NARGEOT Joël, LEBLEU Bernard et al.	[443]
121	US20010018416	2001.08.30	US	Others	Polypeptides	Compositions and methods for treating hepatitis-c	SLESAREV Vladimir I, DIMITROV Todor	[444]
122	US20060089491	2004.06.07	US	Cancer	Polypeptides/nucleotide complexes	Fas-ligand derived polypeptides	NAGATA Shigekazu, SUDA Takashi, TAKAHASHI Tomohiro, NAKAMURA Norio	[445]
123	US20090169599	2009.07.02	US	Others	Reprogrammed virus	Scientifically modulated and reprogrammed treatment (smart) fas/fasl virus technology intended to neutralize t-helper cells infected with the human immunodeficiency virus	SCHEIBER Lane Bernard SCHEIBER II Lane Bernard	
124	EP2355833/US20100324116	2011.08.17/2010.12.23	US/EP	Cancer	RNA interfering molecules	Fas/fasl or other death receptor targeted methods and compositions for killing tumor cells	KRUSE Carol, TRITZ Richard	[283]
125	US20050119212	2004.06.18	US	Autoimmune diseases/others	RNA interfering molecules	Rna interference mediated inhibition of fas and fasl gene expression using short interfering nucleic acid (sina)	HAEBERLI Peter, MCSWIGGEN James	[446]
126	US20070004666	2007.01.04	US	Cancer/autoimmune diseases/others	Transcription factors	Methods for modulating apoptotic cell death	LASHAM Annette, WATSON James D	[447, 448]
127	WO1998008965	1998.03.05	WO	Autoimmune diseases/others	Transcription factors/nucleotide complexes	Cd95 regulatory gene sequences and transcription factors	WATSON James, D, RUDERT Fritz	[447, 448]

The source of information used is the Patentscope of the WIPO IP Portal and the search was carried out by selecting only the patents published in the United States Patent Office and European Patent Office. Furthermore, the selection was made using keywords and selecting only the patents with these keywords in the title of the publication or the corresponding abstract on the front page. The keywords used are CD95, CD95L, CD95 ligand, Fas, FasL, Fas ligand.

It is now well established that the apoptotic signal is often defective in cancer and that the CD95/CD95L interaction is involved in the tumor cells’ escape from the immune surveillance system. For instance, some tumor cell types [221], i.e., some cancer cells, effector T cells (CD8^pos^), regulatory T cells (CD4^pos^, CD25^pos^), tumor endothelial cells, Myeloid-derived suppressor cells (MDSC), Monocyte-derived human macrophages (MDM), Cancer-associated fibroblasts (CAF), Cancer stem cells (CSC), are able to express CD95L at the membrane, thus conferring the tumor environment a “counterattack” mechanism involved in the elimination of tumor-infiltrating lymphocytes and prevention of successful immunotherapy [222–228]. Interestingly, it was observed that the vascular endothelial cells of some solid tumors also express the membrane-bound form of CD95L through a mechanism involving tumor-derived vascular endothelial growth factor A (VEGF-A), interleukin 10 (IL-10) and prostaglandin E2 (PGE2) [229]. CD95L expression becomes here a defense barrier against CD8^pos^ T cells, preventing their extravasation and their access to the tumor nest [230]. Furthermore, it has been observed that different types of cancer cells release vesicles called Tumor-Derived Exosome (TEX) into the microenvironment, which act as messengers between cells. TEX can carry several immunosuppressive molecules, including membrane-bound CD95L. This represents a further defense mechanism by the tumor cells against the CD8^pos^ T cells that manage to infiltrate the tumor nest [231]. TEX can inhibit the proliferation of CD8^pos^ T cells by apoptotic induction, thus playing a major role in immune evasion [232, 233]. In this context, engineered exosomes appears interesting to design potential immunotherapies such as cancer vaccines [234]. Moreover, inhibition of CD95L could prevent cancer resistance to radiotherapeutic or immunotherapeutic treatments, thus representing another path to follow in cancer immunotherapy. Today it is possible to predict, assess and monitor whether a subject with cancer is sensitive to treatment with immunotherapeutics. The Gustave Roussy institute has published a method and kits to determine if in a sample of a said subject one or more biomarkers, including CD95^pos^ CD4^pos^ T cells, CD95^pos^ CD8^pos^ T cells is present/absent together with its expression level (WO2017140826). In 2019, the soluble metalloprotease-cleaved CD95L, associated with a large number of immune infiltrate cells, has been identified as a possible biomarker for tumor immune infiltration (CD3^pos^ and CD8^pos^, and also CD4 and FoxP3 T cells) in advanced HGSOC (High-Grade Serous Ovarian Cancer) [235]. These biomarkers can facilitate the identification of cancer patients prone to respond or resist to proposed immunotherapy and therefore to select an appropriate and personalized chemotherapy treatment. In the last 20 years, the strategies adopted in cancer immunotherapy can be classified into the two large families of active and passive immunotherapy.

### Active approaches

Active immunotherapy is based on the principle that the drug stimulates the patient’s immune response against the tumor, thus acting indirectly. On the contrary, in the case of passive immunotherapy, the drug is directly capable of destroying the tumor cell. Among the active forms of immunotherapy recognized by the US Food and Drug Administration (FDA) and the European Medicines Agency (EMA), should be mentioned the immunomodulatory mAbs, which mostly inhibit the immunosuppressive receptors expressed by activated T cells (e.g., Ipilimumab inhibiting CTLA-4 and Pembrolizumab inhibiting PD-1), the immunostimulatory cytokines, generally used as adjuvants of other anticancer immunotherapies (e.g., IL-2/Proleukin + Ipilimumab), the immunogenic cell death (ICD) inducers, which exert their antitumoral effect through cytostatic and cytotoxic mechanisms.

#### Immunomodulatory mAbs

In some pathological conditions, the so-called immune system checkpoints act directly as a “brake” in the immune response against cancer. The role of immunomodulatory monoclonal antibodies mAbs is precisely to lift these inhibitions by “removing the brake” of the immune system. To date, the most common and most widely used are the inhibitors of checkpoint Cytotoxic T Lymphocyte-Associated Antigen-4 (CTLA-4), Programmed Death 1 (PD-1) and PD-L1 [236]. There are six drugs targeting PD-1 or PDL-1 and only one targeting CTLA-4 currently approved for use in therapy of different types of cancer. Recently, the combination of two inhibitory checkpoints (i.e., ipilimumab (anti-CTLA-4 and nivolumab (anti-PD-1) has also joined the list of approved drugs, showing good therapeutic efficacy in several studies and thus paving the way for new clinical trials in different types of cancers [237]. However, other immune checkpoints are targeted in preliminary stages of clinical development [238]. In recent years, bispecific antibodies have also been developed with the aim of targeting multiple checkpoints simultaneously (e.g., CTLA-4 and PD-1), thus amplifying the signal [239]. However, this double-targeting has so far showed a higher toxicity as compared to its corresponding single therapies. Some of these double-targeting systems will be described in later sections of this review.

#### Immunostimulatory cytokines

With a counterbalancing action, immunostimulatory cytokines act instead as “stimulators” of the immune response. Cytokines can promote the activation, proliferation and survival of lymphocytes (T, B, NK) so as to obtain an antitumor response. Interleukins, interferons and chemokines belong to this large family. The protagonists in the field of immuno-oncology are certainly interleukin-2 (IL-2), the first cytokine FDA approved for therapeutic purposes, IL-12, -15, -21, and interferon alpha (IFN-ɑ), for a long time used for the treatment of hematological neoplasms, for renal carcinoma and melanoma [240]. Having a short half-life, the efficacy of these drugs is limited following their systemic administration. They also induce severe adverse effects before reaching therapeutic doses [241–243]. Today the new engineered generation of these cytokines is making its way into the world of oncological immunotherapy, with improved half-life, antitumor efficacy and toxicity [244].

#### Immunogenic cell death inducers

One of the most widely used ICD agents is Doxorubicin (DOX), a drug discovered in the late 1960s that acts as a DNA intercalating agent and induces apoptosis. As above-mentioned, it has been observed by several groups that some tumor cell lines express CD95L on their surface [245–249] and more importantly, that DOX-induced apoptosis is mediated by the expression of CD95L with the consequent induction of cell death by binding to CD95 [250]. This observation introduced a new perspective on the use of the targeted CD95/CD95L system. In the following years, several other cytotoxic drugs showed the ability to up-regulate the expression of CD95L in cancer cells. In addition to doxorubicin, among the many, we mention Cisplatin, Etoposide, 5-Fluorouracile, Methotrexate, and Bleomycin [251–253]. A parallel mechanism by which these DNA-damaging chemotherapy agents lead to autocrine or paracrine apoptosis of the cell involves the activation of the p53 system, which once activated acts as a transcription factor that regulates the expression of pro-apoptotic genes such as PUMA and BAX [254, 255]. Several studies have been conducted on the implication of p53 in the regulation of dose-dependent heavy side effects, the first of which is cardiotoxicity [256, 257]. Other studies explored the combination of DOX with different drugs with the aim to reduce both acute and chronic DOX-induced cardiotoxicity without affecting its p53-mediated anticancer activity. These studies mention beta-blockers (e.g., Carvedilol), iron-chelating agents (e.g., Dexrazoxane, DEX), angiotensin-converting enzyme inhibitors -ACEI- (e.g., Zofenopril) or even Flavonoids (e.g Frederine), used in combination with DOX for the attenuation of cardiotoxicity [258–261]. Very recently, Todorova et al. carried out a study in which a new combination of DOX and Dantrolene is proposed. Dantrolene appears to mitigate the cardiotoxicity of DOX without affecting its antitumor action in a breast cancer model [262]. Also in 2020, a team from China has developed a new method of co-administration of the DOX, according to which by pre-treating the triple-negative breast cancer cells MDA-MB-231 with Quercetin, followed by the DOX, it is possible to hinder the multidrug resistance of this aggressive cell line [263]. Taken together, these observations are promising for future development from a clinical point of view.

#### Cancer vaccines

The concept of cancer vaccines was first introduced in the 1990s when Bacillus Calmette-Guerin was approved by the FDA for the treatment of early-stage bladder cancer. To date, only three cancer vaccines have been approved by the FDA, due to the poor results often obtained in phases III and IV of the trials [264]. The cancer vaccine approach does not aim to prevent the cancer onset but to activate the immune system against cancer cells. The patent WO2015197874, published by a German team in 2017, proposes a combination of inhibition of the CD95/CD95L complex and cancer immunotherapy, such as a cancer vaccine [265–268]. As previously mentioned, CD95L expression on the tumor endothelium promotes an immunosuppressive environment through preferential killing of tumor-reactive CD8^pos^ cells. Thus, the cancer vaccine would try to get the immune system to mount an attack against cancer cells by using a simultaneous inhibition of the CD95L/CD95 signaling system. More specifically, this cancer vaccine would contain cancer antigens in the form of a protein, a fragment thereof, or as RNA or DNA encoding that protein, to stimulate the immune system against this antigen.

### Passive approaches

#### Monoclonal antibodies

Passive forms of immunotherapy include mAbs that specifically target the receptors on the surface of neoplastic cells expressing “tumor-associated antigens” (TAA), by altering their functions. Some of these antibodies can be administered in combination with chemotherapeutic agents so that the antibodies deliver these agents specifically to cancer cells. An example of such a system is represented by the combination of anti-CD95 antibodies with a chemotherapeutic agent such as 5-Fluorouracil or Tomudex (WO2003097698) [269, 355]. The goal is to synergize the pro-apoptotic effect of anti-CD95 mAbs with cancer chemotherapeutic agents to kill cancer cells. A different combination approach consists in genetically fusing a full-length monoclonal antibody targeting the cancer cell, such as rituximab, with a more biological component represented by a TNF superfamily ligand, such as CD95L, in its full-length, or truncated form, or a fragment thereof, thus offering two approaches to kill cancer cells. The antibody-TNFSF ligand fusion molecules would combine the specificity of the antibodies to the target antigen with the potent death-inducing properties of the TNFSF member ligand, thus providing improved efficacy and safety. The two combined killing approaches are thus performed through ADCC-independent apoptosis (Ab-dependent cellular cytotoxicity), and the second through the recruitment of effector cells to kill tumor targets (WO2012170072) [270]. Another technique involves the use of bispecific antibodies, the method of which consists of linking an antibody that reacts with the tumor cell to a second antibody that reacts with a cytotoxic effector cell. This is the case of patent WO2014076292 published by Baliopharm AG concerning a bispecific antibody with a first binding site for the CD95 receptor and a second binding site for the CD20 antigen [271, 272, 351]. This strategy aims to improve the treatment carried out with rituximab, an antibody able to target and kill CD20 expressing malignant and normal B cells unspecifically, thus showing significant side effects. This technique brings the effector cell into close opposition to the tumor cell, producing increased tumoricidal activity. Overall, the results of preclinical tests performed on antibody systems referring to CD95 have been encouraging, but to date, none of these CD95-related molecules are currently in clinical trials.

#### ACT adoptive cell transfer

Adoptive cell transfer is another very promising type of passive immunotherapy, which involves the re-introduction of specific effector cells into the patient bloodstream [273, 274]. Among them, Lymphokine Activated Killer Cells (LAK), Tumor-infiltrating lymphocytes (TILs) and Chimeric Antigen Receptors (CAR)-T cells are to be mentioned. Lymphokine Activated Killer Cells are obtained from the patient’s endogenous T cells, which are extracted, cultured in the presence of the lymphokine interleukin-2 (IL-2) and reinfused into the patient’s blood [275]. Tumor-infiltrating lymphocytes may have greater tumoricidal activity than LAKs, as they are isolated from resected tumor tissue, thus originating cells with greater tumor specificity than those obtained from blood [276]. An interesting method has recently been published by Iovance Biotherapeutics, Inc., concerning the expansion of TILs from tumor cells using, among others, CD95 agonists, for the treatment of diseases such as cancer (WO2018129332) [277, 278, 366]. A more recent strategy has been developed on the idea of genetically modifying T cells to express TAA-specific T-Cell Receptors, or Chimeric Antigen Receptors that recognize specific proteins on the cancer cells surface. Lately, it has been shown that CAR-T cells up-regulate the expression of CD95 and CD95L resulting in activation of the cell death program independently of TCR or CAR antigen-mediated activation [279]. The work of the Donda’s team highlights the importance of the role of the CD95/CD95L system in CAR-T cells-induced apoptosis by demonstrating the rescue of CAR-T cells upon in vivo blockade of this death-signaling pathway by CD95-Fc recombinant proteins. Patent US20180008670, published in 2018 in the U.S., concerns a method using CAR-T cells to stimulate immunity towards tumor endothelial cells. It is known that one of the limitations of CAR-T cells includes the lack of ability for the T cells to infiltrate deep into tumor tissue. In this formulation CAR-T cells would be able to destroy the CD95L-positive tumor endothelial cells, but also survive in their presence [280]. A year later an American group made the observation that CD95 is highly expressed on patient-derived T cells used for clinical ACT (adoptive cell transfer). They elaborated a T-cell co-engineered system including CD95 DNR (Dominant Negative Receptor) and either a T-cell receptor or Chimeric Antigen Receptor. These cells were genetically modified to express a defective CD95 variant, impairing the induction of apoptotic signal, together with a Chimeric Antigen Receptor, resulting in superior antitumor efficacy, greater longevity and no observed autoimmunity [281]. An interesting observation was recently made by Joshua D Brody’s team, who found that the CD95/CD95L system, in addition to its antigen-specific T-cell killing capability, mediates off-target “bystander” killing of antigen-negative tumor cells. They propose that CD95-mediated bystander elimination of Ag-loss variants may already be occurring in CAR-T treated patients. This process appears to be induced by CD95 upregulation on tumor cells after exposure to T-cell-secreted IFN-γ. They developed a CAR-T mouse model showing an improvement in tumor clearance when CD95 signaling is intact [282]. Overall, these observations open the door to promising new therapeutic opportunities exploiting the CD95/CD95L system in the cancer immunotherapy context

## Therapeutic perspectives in cancer

It is well described that CD95 can promote pro-apoptotic and anti-apoptotic activities according to the physiological context [90]. Some previous studies have shown that down-regulating CD95 via shRNA in cancerous cells activates a death program by the induction of DNA damage and the activation of apoptotic effectors. One of these studies has been carried out and exposed in the US20100324116 patent, in which the inventors set out a siRNA-agent with the aim to reduce the amount of RNA encoding a CD95/CD95L gating polypeptides (e.g., FAPP2 or PATZ1 polypeptides) in significant quantities to sensitize brain tumor cells to CD95-mediated apoptosis [283, 284]. Over the past 25 years, several different potential therapeutic strategies related to the CD95/CD95L interaction have been studied. Among them, polypeptide systems, fusion proteins, methods, chemicals, antibodies and drug delivery systems are probably the most extensively studied.

### Antitumoral polypeptides

Few patents describing CD95-related polypeptides have been published to provide a different approach in cancer treatment. In 2015 it was observed that blood polymorphonuclear neutrophils (PMNs) could kill cancer cells with a mechanism that remains to be elucidated [285]. Last year, a new method for reducing the toxicity of anticancer treatment on normal or non-cancerous cells has been registered as a patent at the University of Chicago (WO2020132465). This invention showed that ELANE, identified as the major anticancer protein released by PMNs, could cleave the CD95 receptor, releasing an intracellular proteolytic fragment containing the Death Domain and selectively killing a wide range of cancer cells [286]. The invention is a combination of specific CD95 peptides and the DNA encoding these peptides to treat different types of cancer. As previously mentioned, metalloproteases-cleaved CD95L (sCD95L) can exert a pro-oncogenic activity, through its interaction with CD95, promoting the survival and proliferation of cancer cells, but also their dissemination [141]. Therefore, a group proposed (WO2015158810) the use of polypeptides composed of the amino acid sequence encompassing the intracellular domain of CD95 which they previously identified as inducing a calcium-dependent cell motility process in T lymphocytes [145]. These inventors reported that the use of such peptides prevented the activation of PLCγ1 and the consequent calcium response that leads to cell migration. The same group reported a few years later that five molecules selected from the FDA/EMA-approved chemical library, namely Ritonavir, Diflunisal, Anethole, Rosiglitazone and Daunorubicin, could all block the recruitment of PLCγ1 to CD95 and reduce T lymphocyte motility (WO2018130679). Less recently, Wagner and Wei published a method related to the use of a combination of polypeptides and their encoding polynucleotides (WO2008067305) [287, 288]. They proposed a polypeptide composed of a ligand domain for a stimulatory Natural Killer receptor (e.g., the extracellular domain of MULT-I, which binds the NK cells receptor NKG2D) and the CD95 intracytoplasmic death domain. This method is supposed to activate the NK cells through the NKG2D receptor after contact with the tumor cells expressing the polypeptidic fusion compound so that not only the engaged tumor cells will be killed via CD95 induced-mechanisms but also are lysed directly by the activated NK cells.

### CD95-related chimeric proteins

Another similar therapeutic approach related to the CD95/CD95L system for the treatment of cancer is the use of fusion proteins or chimeric proteins. Since the years 2000s, the fusion protein system has been perhaps the most widely studied. Patent WO2014121093 should be mentioned among the most recent of them [289]. Here, the inventors elaborated a chimeric system composed of a component capable of inducing the CD95-mediated apoptotic signal, and a component capable of blocking the CD47 receptor expressed at the tumor cell membrane and involved in the suppression of macrophage phagocytosis of the tumor cells.

The approach concerning fusion proteins that provide a physiologically similar oligomerized form of CD95L was studied by two different groups. One exposed a bi-component protein comprising the CTLA-4 extracellular domain and the CD95L extracellular domain, present in the form of a covalently bound and stable homo-hexamer, suitable for the treatment of a patient with cancer (WO2014106839) [290, 291]. If said patient has a tumor expressing the B7 receptor (e.g., B-cell lymphoma), this compound should be administered to exploit the double affinity of the bi-protein for the B7 and CD95 receptors, and finally inducing apoptosis of the malignant cells. The second group instead describes a chimeric protein composed of the extracellular domain of CD95L and a domain capable of inducing the oligomerization in this chimeric system (WO2013060864) [292–294]. Said domain is represented by the Ig-like domain of the Leukemia Inhibitory Factor (LIF) receptor gp190, which self-associates in the context of the chimeric protein giving rise to a dodecameric form with cytotoxic activity towards the cells expressing CD95. This system could therefore have various applications in the clinical field for the treatment of various diseases, such as cancer, autoimmune diseases and others.

### Innovative antitumoral methods

The innovative methods approached in the context of the treatment of cancer patients are numerous and varied, among the most recent of which are the patents WO2015189236, WO2015104284, and WO2014118317, all filed and published by the same group. The first concerns a method aimed to reduce CD95-induced cell migration (WO2015189236). NHE1 is a NA^+^/H^+^ exchanger channel which this group reported to be indispensable for the CD95-induced cell motility process in fibroblasts [295]. This invention provides pharmaceutical compositions of compounds with NHE1 inhibiting properties to be administered if the subject shows elevated blood levels of sCD95L. This group also reported that in triple-negative breast cancer (TNBC), the serum level of CD95L could constitute an important parameter for the prognosis of the survival time and/or the relapse-free survival time. The same group therefore patented the invention WO2015104284, which aims to first determine the expression of sCD95L in the serum of subjects with triple-negative breast cancer (TNBC) and then to compare this level of expression to a predetermined standard value. The concluding step involves the administration to said subject of an effective therapeutic dose of plasma membranes structural components. The goal is to reduce the fluidity of the plasma membrane, a factor that this group reported as involved in the induction of cell migration by CD95 [296]. Subsequently, this same research group developed another patent, this time concerning the prediction and prevention of metastases in TNBC (WO2014118317). The authors describe a method for identifying serum levels of sCD95L in TNBC patients, stating that these patients develop a high risk of relapse if the level of sCD95L is significantly higher than a standard expression level [141]. In the same couple of years, another inventor published a method for predicting the sensitivity of tumor cells for a given treatment targeting inhibition of the CD95/CD95L system (WO2015107105) [297–299]. The invention more specifically concerns the analysis of the methylation levels of a DNA sequence of a gene belonging to this apoptotic signaling cascade obtained directly from a subject suffering from cancer, and consequent observation on the possible responsiveness of said cancer cells to a specific treatment. DNA and histone modifications remain the two major mechanisms of epigenetic regulation of gene expression [300]. Some inhibitors of these mechanisms, such as Decitabine and Vorinostat, are currently in clinical use to inhibit DNA methylation and histone acetylation respectively. An equally important role in the regulation of gene expression is played by the methylation of histone lysine residues through the action of Histone Methyltransferase (HMTase), for which to date only two chemical inhibitors (Verticillin A and Chaetocin) have been generated and found to be toxic in vivo. The Augusta University Research Institute, Inc. has developed a new inhibitor for HMTase SUV39H1 that appears to be useful in activating certain cytotoxic T-cell effectors, such as CD95L, thereby reversing cancer-induced immune suppression and promoting the killing of cancer cells by cytotoxic T cells (US20190084987) [301].

## Currently used therapies and therapeutic perspectives in autoimmune diseases

Despite our growing knowledge of the immunological abnormalities that can lead to autoimmunity, the etiologies of most human autoimmune diseases remain unclear. This is probably because human autoimmune diseases are generally heterogeneous and multifactorial, not only between different diseases but also within the same disease [302]. They can, within a single disease, present a wide variety of clinical manifestations and severity, for instance the propagation speed, the number of affected joints, as well as a vast phenotypic heterogeneity. Besides, autoimmune diseases can clinically manifest long after the autoimmune reactions have been induced. Autoimmune diseases are often characterized by a severe imbalance between pro and anti-inflammatory mechanisms and by a vast diversity of signaling pathways and of cells and cytokines such as interleukins, interferons, and Treg cells that play a crucial role in immune tolerance. In recent decades, enormous progress has been made to identify the mechanisms associated with the activation and inactivation of T cells and to improve techniques based on the study of selective immune suppression in human autoimmune diseases. To date, the techniques used to counteract the mechanisms of autoimmunity are varied and include different peptide analogs, immunosuppressants, anti-inflammatories, monoclonal antibodies, inducers of immune tolerance, therapies targeting certain autoantigens, often used in conjunction with immunosuppressants to reduce their doses. Several groups around the world have carried out studies for which patents have been filed.

### Multiple sclerosis

MS is a chronic autoimmune neurodegenerative disease that affects the central nervous system (CNS). It is characterized by an abnormal reaction of the immune defenses towards certain components of the CNS, damaging myelin and oligodendrocytes [303]. The symptoms are varied but CNS defective functions are frequent, with recurrent remissions and exacerbations. MS is suspected in patients with optic neuritis, especially if the deficits are multifocal or intermittent. In such cases, magnetic resonance imaging (MRI) scans of the brain and spinal cord and cerebrospinal fluid (CSF) analyses are performed, as this techniques allow to exclude other treatable pathologies that can mimic MS [304]. At the moment there is no definitive cure, but numerous therapies are available to modify its course, slowing its progression. The most severe form of MS is undoubtedly represented by relapsing-remitting multiple sclerosis (RRMS). Subjects with RRMS tend to have more brain lesions with widely varying localization and very different symptoms [305]. To date, the diagnosis to confirm the presence of the disease is given by tests resulting positive at least on two areas of myelin lesions in the CNS. These tests are not only painful but also risky and highly expensive. It is, therefore, necessary to develop additional methods for the diagnosis of this disease. The inventors of US20160194714 offer a new method for detecting relapse in RRMS patients using biomarkers, such as CD95L, sirtuin 1 (SIRT1), RGC-32 and IL-21, in a population of cells (e.g., PBMCs, CD4^pos^, CD8^pos^, glial cells, neurons, etc.) [306–309]. They noted a decrease in CD95L, SIRT1, and RGC-32 in relapsing RRMS patients, while an increase in IL-21 occurs. Overall, these four proteins can be used as markers to highlight the activity of this disease.

### Systemic lupus erythematosus

The involvement of CD95L has been extensively studied in different chronic inflammatory autoimmune diseases, such as MS, SLE and RA. Several groups have observed differences in the frequency of the T-helper cells (Th) subgroups in SLE patients versus HCs (Healthy Controls), which also differ in their sensitivity to TCR-mediated cell death [310–313]. This could explain the discordant results on CD95L expression levels in total lymphocytes from healthy donors and patients with chronic inflammatory disease. A few years ago, it was noted that transcription of CD95L is a crucial step for the regulation of T-helper cell death sensitivity. This group found that human Th1 cells express higher mRNA levels of CD95L than Th17 cells. Resistance of Th17 cells to AICD was associated with lower expression of CD95L and overexpression of the anti-apoptotic caspase-8 inhibitory protein (FLIP) [314]. In the mid-2000s, an important role was attributed to these IL-17A and IL-17F producing lymphocytes in the context of autoimmune diseases. Th17 cells orchestrate autoimmune inflammation, in addition to their function as eliminators of extracellular pathogens [315–317]. Yang et al. observed that SLE patients exhibit significant infiltration of Th17 lymphocytes secreting cytokines in their skin [318]. It is therefore possible to hypothesize that by modulating their trafficking to the organs, the pathogenesis of the SLE disease could consequently be modulated. In the context of this chronic inflammatory disease, soluble CD95L (sCD95L) has been shown to be involved in promoting the trafficking of Th17 lymphocytes into damaged organs, at the expense of Treg lymphocytes in a CD95-driven murine model of SLE [142]. Blocking the CD95/CD95L system could thus represent an attractive approach for the treatment of Th17 cell-mediated diseases. This was the intent of the authors of the WO2016170027 patent, who proposed to use CD95 antagonist antibodies, having specificity for CD95 or sCD95L, with the potential to prevent the endothelial transmigration of Th17 cells in the organs and the consequent damage given by the accumulation of the activated T cells in said organs [142]. DR-mediated cell death is essential for the differentiation, growth and function of lymphocytes. In 2017, Croft and Siegel discussed the implication of some of these receptors in inducing inflammation and their potential in future therapies for rheumatoid diseases [319]. Interestingly, the combined blockade of TNFR1, TRAIL-R and CD95 seems to give excellent results in the prevention of inflammation caused by the respective ligands, whereas targeting these receptors individually did not have that effect (WO2019141862) as demonstrated in a murine model of dermatitis [320]. Such observations lead to the conclusion that different cell DR systems may act in combination to contribute to the pathogenesis of autoimmune inflammatory diseases. Importantly, uncontrolled induction of cell death downstream of DR, rather than increased DR-induced gene-activatory signaling pathways, could actually be key in driving inflammation in such contexts [320–322]. Interestingly, the Decoy Receptor 3 (DcR3), encoded by the *TNFRSF6B* gene, was found to act as a regulator of the amplification of the immune response by binding with stimulatory cytokines, such as CD95L, TL1A and LIGHT, limiting the interaction of the latter with their own receptor [323]. It, therefore, seems deductible that genetic modifications of the *TNFRSF6B* gene, involving a reduced expression of DcR3, or a lower binding activity for the aforementioned cytokine, or even the suppression of its expression, could contribute to cause inflammatory signals. With this idea in view, the inventors of US20170051352 have developed a method for treating autoimmune conditions in patients carrying alterations of the gene encoding the DcR3 protein, or of a DcR3 network gene, by administering to said patient an effective amount of DcR3 ligands inhibitors [323].

### Fusion proteins in the context of autoimmune diseases

As in the context of cancer, one of the widely adopted strategies in studying new potential treatments for autoimmune diseases is represented by the use of fusion proteins and nucleotides that encode them. In 2018, APOGENIX AG published a patent relating to a nucleotide sequence encoding an isolated chimeric compound formed by the extracellular domain of CD95 and an immunoglobulin domain or a functional fragment thereof. The inventors intend to generate a stable system to inhibit the extrinsic apoptotic signal initiated by CD95L for the prophylaxis or treatment of various diseases, including autoimmune diseases and solid cancers (US20180186856) [324, 325, 402]. A few years earlier the same inventors developed a mixture of fusion protein isoforms having the same composition as the aforementioned system with the difference that this patent does not mention any nucleotide sequence encoding the chimeric protein, as well as the cell hosting the nucleotide sequence (WO2014013039). A different chimeric system is represented by the invention WO2016205714, which exposes an immune tolerance inducer “medicament” comprising a CD95L moiety together with a streptavidin or avidin moiety [326–328]. The claimed compound is to be administered alone or mixed with the IL-2 protein to achieve sequential or simultaneous action in inducing long-term and specific immunosuppression. CD95L is then part of another fusion compound, the one described by the patent WO2014121085, in which the extracellular domain of CD95L corresponds to half of the fusion protein. The other half is the extracellular domain of a PD-1 receptor-activating factor, such as its ligand PD-L1 and PD-L2 [329]. This system aims to inhibit the differentiation and proliferation of a selection of cells, including activated T cells on which the PD-1 receptor is widely expressed, thus the induction of PD-1 ligation by its ligands mediates an inhibitory signal that results in reduced cytokine production and reduced T-cell survival. Thus, in the setting of autoimmune and inflammatory diseases, the fusion protein of this invention could reduce autoimmune and inflammatory manifestations.

### Cells engineering modifications

Some other groups have then explored the field of cells engineering modification by proposing methods of isolating these cells from a patient sample, treating/modifying these cells and reintroducing the said modified cells by systemic infusion or transplantation. This is the case of the patent WO2013149211, which describes a method using modified mesenchymal stem cells (MSCs) to overexpress CD95, CD95L as well as the CD95-regulated monocyte chemotactic protein 1 (MCP-1) which seems to play an important role in the recruitment of T cells to MSCs [330, 331]. It has previously been hypothesized that such MSCs play an important role in reducing T-cell proliferation through a mechanism involving T-cell apoptosis [332]. Therefore, this invention offers a potential therapeutic method for the treatment of autoimmune diseases, and more specifically of Systemic Sclerosis. Similarly, patent WO2015038665 relates to a system composed of modified MSCs to overexpress CD95L after exposure of these cells to a salicylate, such as common aspirin [333]. The authors offer a method aimed at increasing survival rates in patients suffering from autoimmune and inflammatory diseases. In 2016, another modified cell-related strategy was developed by the Trustees of the University of Pennsylvania, which involves the use of genetically modified effector cells to downregulate endogenous CD95 using the CRISPR system to treat autoimmune diseases (WO2016069282) [364].

## Discussion and conclusion

For nearly three decades, members of the TNF superfamily, and the signal cascades they trigger, have been targeted by researchers and pharmaceutical companies to develop new therapies for the treatment of cancer and autoimmune diseases [334–338]. These molecules are widely involved in multiple cellular mechanisms such as apoptosis, proliferation, survival, tumor growth and differentiation. Since their role in mediating immune surveillance as well as protection from infections is essential, prolonged inhibition of these molecules could be dangerous. The progenitor of the TNF superfamily (i.e., TNF) remains the most studied and the most promising in terms of therapeutic potential [337, 338]. Among the members of the TNF superfamily, the research carried out on the TNF system is the most funded, with sales revenues exceeding 25 billion USD [338] followed by DR4/DR5 (Trail) systems and finally by the CD95 complex. Currently, five anti-TNF biologics have been clinically approved for the treatment of autoimmune diseases, namely Infliximab, Adalimumab, Etanercept, Golimumab, and Certolizumab Pegol, all with a specific structure for TNF-alpha recognition and blockade [339]. Despite the evident efficacy of these drugs, not all treated patients respond as expected and some seem to develop adverse reactions associated with these drugs, such as effects on the neurological and dermatological levels [340–342]. There is therefore a growing need for new pharmacological systems with better specificity and greater safety.

### CD95-related therapeutic perspectives

**Fig. 4:**
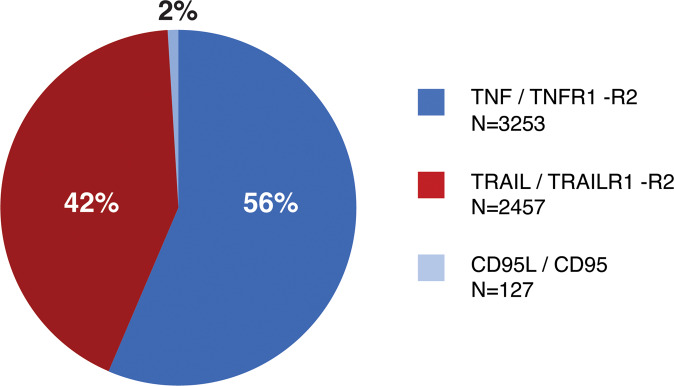
Contribution of the CD95L/CD95 system in therapeutic-end studies. Graphic representation of the distribution of the number of patents targeting the most studied cell Death Receptors CD95, TNFR1 and -R2, TRAILR1 and -R2 and their respective ligands CD95L, TNF alpha, and TRAIL.

Despite the evident role of CD95/CD95L in cancer and chronic inflammatory autoimmune diseases, since 1990 only a little over a hundred patents targeting the CD95/CD95L system have been conceived and published (Fig. 4). In the past, the complexity of the multiple CD95/CD95L-mediated signaling systems found in cancer and autoimmune diseases, the lack of specificity of the previously proposed strategies tested in vivo and the consequent severe side effects found [219, 220], have diminished the pharmacological interest for this target. Recent study report strategies focusing on more challenging compounds and delivery methods, with a particular attention to circumventing the severe adverse effects associated with the systemic activation of CD95. The extensively studied CD95-Fc fusion proteins, for instance, represent an interesting way to inhibit CD95L. However, these chimeric proteins, compared to those used in the TNF-TNFR2 system [343], exhibit a relatively low affinity for the corresponding CD95L and far less efficacy in inhibiting death induced by ligand interaction with CD95. A possible explanation is given by the fact that the interactions between these proteins occur through a complex mechanism of oligomerization given by the association of multiple trimers of both counterparts [35, 294, 344]. A better neutralization or stimulation of these proteins might therefore be achieved by a neutralization/stimulation system in which the binding protein is in a stable physiological-like form consisting of at least one trimer, if not an oligomer thereof. It seems that the oligomerization of the binding protein improves the stability of the therapeutic compound, consequently increasing its affinity for the target and the final system specificity [345]. Such oligomerized-related strategies have exhibited more efficient results, compared to previous systems generations. Fortunately, some of the newly proposed strategies appear to give encouraging preclinical results and so far, only one of these is currently in clinical trials. APG101 is the best prototype of future therapeutic approaches involving the CD95 system. It is an 84 kDa CD95L-neutralizing CD95 trimer fusion protein, able to pass the blood-brain barrier. Asunercept, the trade name for APG101, is now the subject of a controlled phase II clinical trial in patients with relapsed glioblastoma multiforme (GBM) (NCT01071837). The glioblastoma model was chosen in accordance with several in vivo and in vitro non-clinical studies, which extensively described the involvement of CD95L in the growth, invasiveness and migration of glioblastoma cells [152, 324]. Merz et al. observed a decreased invasiveness on two cellular models of GBM after knockdown of the *FASLG* gene, without however affecting the viability of the cells sensitive to apoptosis [325]. They also reported a restored invasiveness following the administration of soluble recombinant CD95L, which was blocked by the addition of the APG101 fusion protein. This formulation, consisting of the extracellular domain of human CD95 and the Fc domain of human IgG1, was in fact designed to specifically bind CD95L, thus disrupting the CD95L/CD95 signal cascade and the resulting cellular invasiveness. The collected results show a remarkable survival prolongation in patients with GBM, which makes it interesting for a possible transfer to other types of cancer [324, 346]. Furthermore, some experiments carried out on a cohort of 84 patients, showed greater efficacy of this compound when it is administered in combination with radiotherapy, observing a significant reduction in tumor growth compared to radiotherapy treatment alone [347]. Other preclinical studies, conducted on patients suffering from a lower risk myelodysplastic syndrome (MDS), have then highlighted a possible role of Asunercept in the treatment of anemia, a characteristic feature of this pathological condition [348]. In low risk MDS the administration of erythropoiesis-stimulating agents (ESAs) is widely used to correct cytopenia. However, some patients show resistance to ESA, thus requiring alternative treatments to contain the anemia associated with low risk MDS. CD95 is overexpressed in two-thirds of MDS patients, and is thought to be negatively implicated in the regulation of erythrocyte production [348]. The blocking of CD95 signal cascade can therefore increase erythropoiesis in MDS patients. The use of APG101 in this context seems to be particularly promising, as the neutralization of CD95L allows the blocking of the CD95L/CD95L signal and finally the restoration of erythropoiesis. A phase I clinical study (NCT01736436) conducted on 20 patients with low and intermediate MDS treated with intravenous APG101 is currently underway [349]. In said patients APG101 showed good tolerance and safety, promising prerequisites for use on a larger scale of this drug in the future.

## Supplementary information

Reproducibility checklist form

## Data Availability

All data generated or analyzed during this study are included in this published article and Supplementary Materials.
